# Supplementary material for: A multicentral prospective cohort trial of a pharmacist-led nutritional intervention on serum potassium levels in outpatients with chronic kidney disease: The MieYaku-Chronic Kidney Disease project
Source: PLoS One. 2024 May 31;19(5):e0304479. doi: 10.1371/journal.pone.0304479 (PMC11142692; doi:10.1371/journal.pone.0304479)
Supplement: S1 File — (DOCX) [file pone.0304479.s003.docx]

**Study protocol**

**Evaluation of efficacy of the community pharmacy intervention to optimize serum potassium levels in patients with chronic kidney disease**

　　Abbreviation: Mieyaku-chronic kidney disease: My-CKD trial

Principal Investigator: Pharmacy, Mie Chuo Medical Center, National Hospital Organization

Yuki Asai

Research Secretariat: Pharmacy, Mie Chuo Medical Center, National Hospital Organization

Yuki Asai

Created on November 4, 2022 Ver 1.0

Created on November 24, 2022 Ver 2.0

[Creation and revision history]

| Version No. | Created/Revised Date | Reason for revision |
| --- | --- | --- |
| Ver.1.0 | November 4, 2022 | New |
| Ver. 2.0 | November 24, 2022 | Revision after the conclusion of the Ethics Review Committee |
|  |  |  |
|  |  |  |
|  |  |  |

**table of contents**

[0. Shema 1](#_Toc120209233)

[0.1. Summary of study design 2](#_Toc120209234)

[1. Research Implementation Structure 3](#_Toc120209235)

[1.1. Principal Investigator 3](#_Toc120209236)

[1.2. Research Secretariat 3](#_Toc120209237)

[1.3. Principal Investigator 3](#_Toc120209238)

[1.4. Co-Investigators 4](#_Toc120209239)

[1.5. Head of Statistical Analysis 4](#_Toc120209240)

[1.6. Head of Data Management 4](#_Toc120209241)

[1.7. Personal Information Manager 4](#_Toc120209242)

[1.8. Person in charge of monitoring 4](#_Toc120209243)

[1.9. Audit Officer 4](#_Toc120209244)

[2. Purpose and Significance of Research 4](#_Toc120209245)

[2.1. purpose 4](#_Toc120209246)

[2.1.1. Primary Objective 4](#_Toc120209247)

[2.1.2. Secondary Objectives 5](#_Toc120209248)

[2.2. background 5](#_Toc120209249)

[2.3. Significance of the study 5](#_Toc120209250)

[3. Method and duration of the study 5](#_Toc120209251)

[3.1. Study design (research method) 5](#_Toc120209252)

[3.2. Number of Planned Research Subjects and Rationale for Establishment 7](#_Toc120209253)

[3.2.1. Number of Planned Research Subjects 7](#_Toc120209254)

[3.2.2. Rationale for setting the number of planned research subjects 7](#_Toc120209255)

[3.3. Research Period 7](#_Toc120209256)

[3.4. data collection 7](#_Toc120209257)

[Forms and Submission Deadlines 7](#_Toc120209258)

[How to fill out 7](#_Toc120209259)

[How to send 8](#_Toc120209260)

[3.5. Methods of statistical analysis 8](#_Toc120209261)

[3.5.1. Population to be analyzed 8](#_Toc120209262)

[3.5.2. Statistical analysis 8](#_Toc120209263)

[3.5.3. Subpopulation analysis 8](#_Toc120209264)

[3.5.4. Interim Analysis 9](#_Toc120209265)

[3.6. Observation items and methods 9](#_Toc120209266)

[3.6.1. Observations and information and samples to be collected 9](#_Toc120209267)

[3.6.2. Observation and Reporting Schedule 10](#_Toc120209268)

[3.6.3. Collection and Evaluation of Adverse Event Information 10](#_Toc120209269)

[3.7. Discontinuation and termination of the study 10](#_Toc120209270)

[3.7.1. Discontinuation of study subjects 10](#_Toc120209271)

[3.7.2. Discontinuation of the entire study 10](#_Toc120209272)

[3.7.3. Termination of study 11](#_Toc120209273)

[4. Selection Policy for Research Subjects 11](#_Toc120209274)

[4.1. Eligibility Criteria 11](#_Toc120209275)

[4.1.1. Criterias of choice 11](#_Toc120209276)

[4.1.2. Exclusion Criteria 11](#_Toc120209277)

[4.2. Recruitment of research subjects 11](#_Toc120209278)

[4.3. Registration of study subjects 11](#_Toc120209279)

[5. Basis for the scientific rationality of the study 12](#_Toc120209280)

[6. Procedures for obtaining informed consent, etc. 12](#_Toc120209281)

[7. Handling of Personal Information 14](#_Toc120209282)

[8. Burdens and anticipated risks and benefits to the study subjects 14](#_Toc120209283)

[8.1. Burden and anticipated risks to research subjects 14](#_Toc120209284)

[8.2. Expected benefit to the study subject 14](#_Toc120209285)

[8.3. Comprehensive evaluation of these and measures to minimize burdens and risks 14](#_Toc120209286)

[9. Methods of storage and destruction of samples and information 14](#_Toc120209287)

[10. Contents and methods of reporting to the head of the research institute 15](#_Toc120209288)

[10.1. Reports from Researchers 15](#_Toc120209289)

[10.2. Report from the Principal Investigator 15](#_Toc120209290)

[10.3. Report from the Auditor 15](#_Toc120209291)

[11. Conflicts of interest related to research, such as sources of funding for research 15](#_Toc120209292)

[12. How to disclose information about research 15](#_Toc120209293)

[13. Responding to consultations from research subjects and related parties 16](#_Toc120209294)

[14. Procedures for Obtaining Informed Consent from a Substitute, etc. 16](#_Toc120209295)

[15. Procedure for obtaining an informed ascent 16](#_Toc120209296)

[16. Statement of financial burden or gratuity of research subjects, etc., and their details 16](#_Toc120209297)

[17. Response in the event of a serious adverse event 16](#_Toc120209298)

[17.1. Serious adverse events 17](#_Toc120209299)

[17.2. Predictable adverse events 17](#_Toc120209300)

[17.3. Emergency Reporting 17](#_Toc120209301)

[18. Whether or not there is compensation for health damage caused by the research 18](#_Toc120209302)

[19. Measures to be taken regarding the provision of medical care after the conduct of the research 18](#_Toc120209303)

[20. Handling of research results related to research subjects 18](#_Toc120209304)

[21. Details of outsourcing research-related work and method of supervision of the contractor 18](#_Toc120209305)

[22. If there is a possibility that the samples and information obtained from the research subject will be used for future research that is not specified at the time of obtaining the consent from the research subject, etc., or that may be provided to other research institutions, that fact and the contents that are expected at the time of obtaining the consent 18](#_Toc120209306)

[23. When monitoring and auditing are to be conducted, the implementation system and procedures 18](#_Toc120209307)

[23.1. monitoring 18](#_Toc120209308)

[23.2. audit 19](#_Toc120209309)

[24. other 19](#_Toc120209310)

[24.1. References 19](#_Toc120209311)

[24.2. Appendix 20](#_Toc120209312)

# Shema

- Main eligibility criteria: Patients with an estimated glomerular filtration rate (eGFR) of less than 45 mL/min/1.73 m2 who attend the Mie Chuo Medical Center of the National Hospital Organization
- Timing of patient registration: The first day of the patient's visit to the pharmacy of the cooperating facility during the enrollment period
- Number of cases and registration period: 40 people, December 2022 ~ February 28, 2023
- Intervention Methods:

1. Questionnaire survey on awareness of the intake of potassium-containing foods

2. Dietary guidance: Nutritional guidance using explanatory documents on diet

3. Measurement of resting blood pressure after visit

4. Sharing information on the status of potassium adsorbents with Mie Chuo Medical Center

- Follow-up period: 3 months (after 84 days) from the first day of intervention

Study design

- positive
- Intervention Studies
- Design features: Before-and-after comparison test, open design
- Contrast Type: None
- Facility: Multi-center
- Randomization: No
- Level of Blinding: Open-label


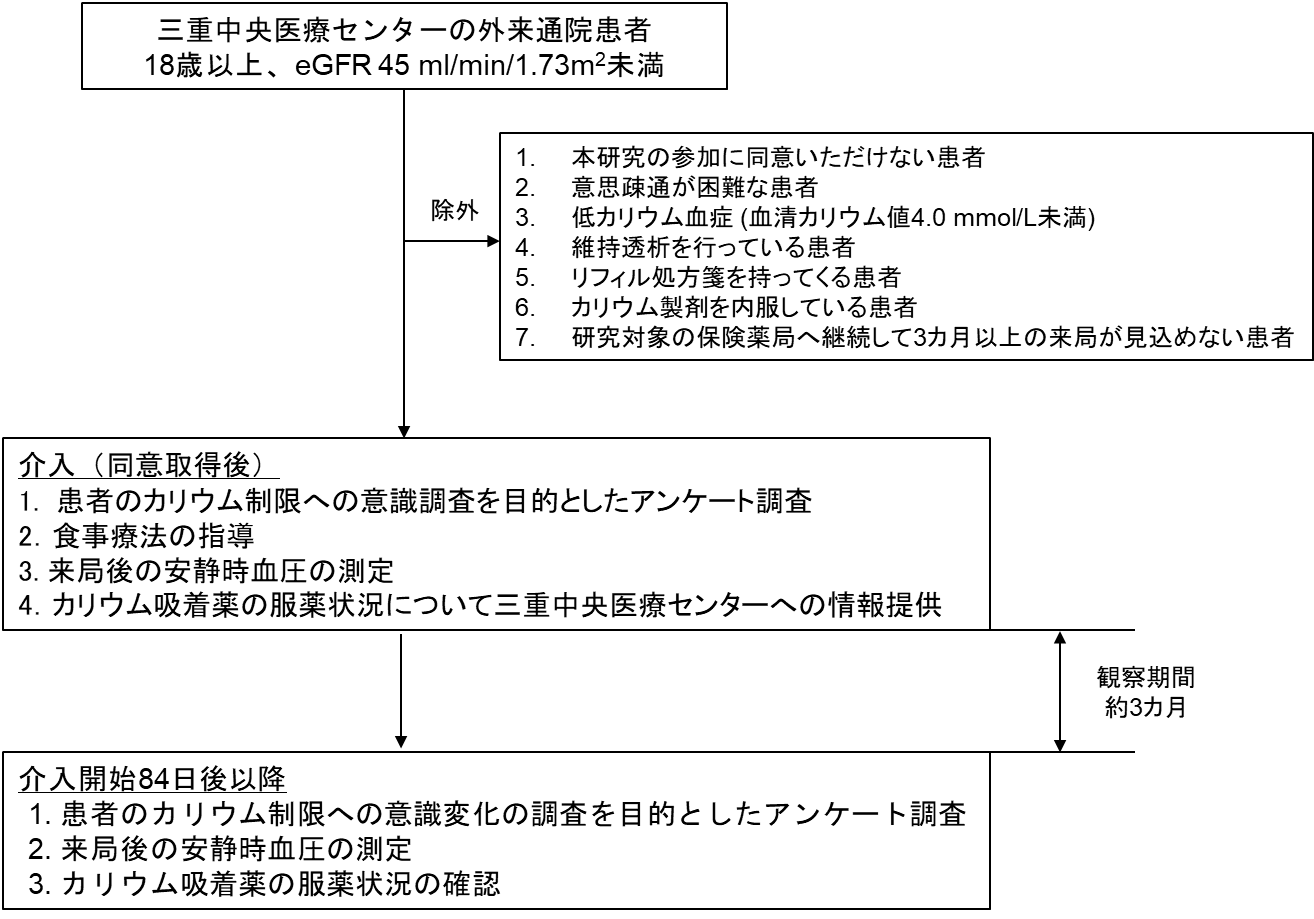


## Summary of study design

Prospective intervention, before-and-after comparison, open design

This research will be conducted in compliance with the Personal Information Protection Law, the Declaration of Helsinki, and the Ethical Guidelines for Life Science and Medical Research Involving Human Subjects.

# Research Implementation Structure

## Principal Investigator

Mie Chuo Medical Center Pharmacy Department Pharmacist Yuki Asai

## Research Secretariat

Mie Chuo Medical Center Pharmacy Department Pharmacist Yuki Asai

(Contact)

Location: 〒514-1101 2158-5 Hisai Myojin-cho, Tsu-shi

Phone: 059-259-1211 (ext. 4724)

FAX　　： 059-256-2651

E-mail ： yuki0715asai@gmail.com

## Principal Investigator

All 23 health pharmacies

Sugi Pharmacy Hisai Intergarden Pharmacist Kaori Miyata

Pharmacy Ipharmacy Myojin Pharmacist Ai Izugawa

Hisai Dispensing Pharmacy Pharmacist Tatsuya Kobayashi

Sugi Pharmacy Hisai Shinmachi Pharmacist Tomoharu Hasebe

Kokoro Dispensing Pharmacy Pharmacist Hiroya Inui

Konan Pharmacy Pharmacist Toshiki Murasaka

Hisai Center Pharmacy Pharmacist Ikuhiro Takasaki

Nanohana Pharmacy Kannonji Pharmacist Chie Suezawa

Tsubame Pharmacy Pharmacist Jun Naito

Penguin Pharmacy Pharmacist Ryo Sato

Ichishi Dispensing Pharmacy / Takano store Pharmacist Yuji Nakagawa

Doremi Pharmacy Pharmacist Yuko Niimi

Hisai Nomura Dispensing Pharmacy Pharmacist Yasuki Ogino

Healthy Pharmacy Myojin Pharmacist Hiroki Sugino

Hisai Shinmachi Pharmacy Pharmacist Ryota Kobayashi

Takachaya Pharmacy Pharmacist Jinro Kinoshita

Sugi Pharmacy Tsu Shinmachi Pharmacist Takahiro Fukuyama

Medimo Dispensing Pharmacy Pharmacist Koji Terada

Flower Pharmacy Ichishi Pharmacist Yuka Urakawa

Cocokara Fine Pharmacy Tarumi Pharmacist Hironori Miyaji

Taiyo Pharmacy Pharmacist Tomohiko Aoki

Akira Pharmacy Pharmacist Taisuke Matsumuro

Ichishi Dispensing Pharmacy / Nakamachi Store Pharmacist Nobuyuki Nakagawa

## Co-Investigators

Mie Chuo Medical Center Pharmacy Department Pharmacist Itsuki Yanagawa

Mie Chuo Medical Center, Department of Pharmacy, Director of Pharmacy Department, Yoshiharu Sato

Mie Chuo Medical Center Department of Nutrition Registered Dietitian Asami Muramatsu

Mie Chuo Medical Center Cardiovascular Medicine Physician Takahiro Okazaki

## Head of Statistical Analysis

Mie Chuo Medical Center Pharmacy Department Pharmacist Yuki Asai

## Head of Data Management

Do not install.

## Personal Information Manager

Mie Chuo Medical Center Pharmacy Department Pharmacist Yuki Asai

## Person in charge of monitoring

Not applicable.

## Audit Officer

Not applicable.

# Purpose and Significance of Research

## purpose

In patients with chronic kidney disease (CKD), in addition to conventional medication guidance, nutritional guidance on potassium restriction for a few minutes by a pharmacy pharmacist will clarify the effect on serum potassium levels.

The primary endpoint is serum potassium level, and the secondary endpoint is resting blood pressure after visit, and change in the patient's awareness of potassium restriction.

### Primary Objective

(1) Changes in serum potassium levels before and after intervention

(2) Changes in awareness of the intake of potassium-containing foods before and after the intervention

### Secondary Objectives

| Secondary Objectives | Evaluation items | Reasons and Validity for Selection of Evaluation Items |
| --- | --- | --- |
| Change in Attitudes Toward the Intake of Potassium-Containing Foods | Survey Results | Evaluated on a 5-point scale for questions about awareness of potassium restriction |
| Change in resting blood pressure before and after intervention | Systolic and diastolic blood pressure at rest at visit | Blood pressure fluctuations are expected due to diet |

## background

As of 2016, approximately 330,000 patients with CKD with severely reduced renal function were reported, and further increases are expected in the future1). Patients with CKD have a variety of adverse events associated with decreased renal function, but hyperkalemia in particular is the most common electrolyte abnormality and can cause arrhythmias and sudden death.

Hyperkalemia is triggered by excessive dietary potassium intake2), so it is important for many patients with CKD to be guided to limit potassium intake. However, during inpatient treatment, the patient shows an appropriate serum potassium level due to strict control of diet and drug therapy, but after discharge from the hospital, it is not possible to maintain diet and adherence to potassium adsorbents, so there are some cases of emergency hospitalization for hyperkalemia.

## Significance of the study

Health insurance pharmacies that contribute to community-based medical care may be able to motivate patients to maintain a diet at home and prevent emergency hospitalization due to hyperkalemia by providing nutritional guidance for potassium restriction. Hyperkalemia may improve the patient's life prognosis due to fatal adverse events.

# Method and duration of the study

## Study design (research method)

・Case registration

CKD severity classification stages G3b, G4, G53^)^

When the outpatient prescription of the Mie Chuo Medical Center is brought to the insurance pharmacy, the written consent of the patient is obtained and registered. The date of obtaining consent shall be Day 1.

・Intervention method

All of the following interventions are performed by pharmacists at insurance pharmacies.

Date of Obtaining Consent (Day 1)

1. Questionnaire of the Awareness Survey on the Intake of Potassium-Containing Foods in Patients (Questionnaire 1) Appendix 2

2. Dietary guidance: Guidance on nutritional guidance for CKD (Annex 1) will be used. (Recommendation of nutritional guidance to the Nutrition Management Office as necessary)

3. Measurement of resting blood pressure after the visit

4. Provision of information to the Mie Chuo Medical Center on the status of the administration of potassium adsorbents: If the cause of poor adherence is the dosage form or dosage, the information will be provided to the Mie Chuo Medical Center of the prescriber in a tracing report.

⇒ hospital pharmacist writes details such as dosage status on the bulletin board of the electronic medical record to the prescribing physician and proposes a dosage change.

Day1-83

1. Dietary guidance (as appropriate)

2. Measurement of resting blood pressure after the visit

3. Confirmation of the status of potassium adsorbents (if information is provided to Mie Chuo Medical Center, confirmation of dosage type change, etc.)

After Day 84 (end date of the observation period)

1. Questionnaire on the Awareness Survey on the Intake of Potassium-Containing Foods by Patients (Questionnaire 2) Appendix 3

2. Measurement of resting blood pressure after the visit

3. Confirmation of the status of potassium adsorbents (if information is provided to Mie Chuo Medical Center, confirmation of dosage type change, etc.)

Contents of the questionnaire

For all questions, set a five-level answer.

At the time of obtaining consent (Day 1): Questionnaire 1

Q1. Do you know that potassium in the blood should not be too high?

Q2. Do you know of any foods that contain a high amount of potassium?

Q3. Are you conscious of refraining from foods that contain a lot of potassium?

Q4. Is it annoying (troublesome) to be aware of potassium intake?

3 months after obtaining consent (after Day 84): Questionnaire 2

Q1. Do you know that potassium in the blood should not be too high?

Q2. Do you know of any foods that contain a high amount of potassium?

Q3. Are you conscious of refraining from foods that contain a lot of potassium?

Q4. Is it annoying (troublesome) to be aware of potassium intake?

Q5. Has this guidance made you more careful about your intake of foods that contain potassium?

Q6. Do you want to continue to take measures against potassium-containing foods in the future?

Q7. Please let us know if there are any points that you have noticed through this guidance.

・Observation period

Calculated from the date of obtaining consent (Day 1) and after week 12 (Day 84)

・Data acquisition

During the observation period, each visit to the insurance pharmacy, the resting blood pressure (systolic blood pressure, diastolic blood pressure) is measured and recorded. The present study will be conducted in a non-randomized manner.

## Number of Planned Research Subjects and Rationale for Establishment

### Number of Planned Research Subjects

40 cases at the joint research facility

### Rationale for setting the number of planned research subjects

Previously, nutritional guidance on a potassium-restricted diet reduced serum potassium levels by up to 0.5 mmol/L4^).^ In general, it has been reported that the standard deviation of serum potassium levels between individuals was about 0.5 mmol/L5^).^ However, Kurita et al.5) report a standard deviation of n = 60-70, and the standard deviation in this study may be larger than previously reported. Therefore, the standard deviation in this study was estimated to be 1.0 mmol/L. Assuming that α = 0.8, β = 0.05, the standard deviation of the serum potassium level in the population is 1.0 mmol/L, and the difference in the mean value due to the intervention is 0.5 mmol/L in the paired t-test, the required number of cases is calculated as n=34. Taking into account the number of dropouts, the goal was n = 40.

During the three-month period from May 1 to July 31, 2022, 1,344 patients (eGFR <45 mL/min/1.73 m2 and serum potassium level of 4.0 mmol/L or higher) at Mie Chuo Medical Center were 1,344, and many of them visited the insurance pharmacy of the joint research institute.

## Research Period

Registration Period: From the date of permission (or the date of publication of the RCT) to February 28, 2023

Study Period: From the date of permission (or the date of publication ofthe J RCT) to May 31, 2023

Duration of participation: 6 months

## data collection

## Forms and Submission Deadlines

In this study, the results of the questionnaire before and after the intervention and the following items using Google Forms will be submitted to the principal investigator. The deadline for submission of the following items using Google Forms is within one week after the end of the patient's observation period.

## How to fill out

In the Google Form, the name of the pharmacy, the study registry number, the date of the intervention, the method of managing the medication at home (self-management, family management, facility management, caregiver management), the most recent meal at home (self-catering, home delivery meals, eating out), whether or not you want detailed nutritional guidance from a dietitian, the descriptive form for resting blood pressure at the time of visit, and the descriptive form for resting blood pressure at the time of visit.

The study registry number will be communicated from the principal investigator to the representative of each site after the patient consents to participate in this study. The study registry number, patient ID, and patient name will be prepared and kept by the principal investigator.

## How to send

For items in Google Forms, use the submission form on the Web. The results of the questionnaire will be sent by fax.

## Methods of statistical analysis

### Population to be analyzed

The main analysis of the primary endpoint and secondary endpoint is the one targeting the Full Analysis Set. In addition, we will also conduct an analysis of the Per Protocol Set to confirm the stability of the analysis results.

(1) Definition of Full Analysis Set

Cases that meet all of the following criteria are considered to be cases.

(1) eGFR less than 45 ml/min/1.73 m2 and serum potassium level of 4.0 mmol/L or more

(2) Patients who have obtained consent for this study at an insurance pharmacy

(2) Definition of Per Protocol Set

　　Patients with FAS who meet all of the following criteria are considered to be FAS.

(1) Patients who obtained the results of questionnaire 2 after 84 days after obtaining consent

(2) Patients with resting blood pressure after visiting the clinic after 84 days after obtaining consent

(3) Patients who responded to a questionnaire about their awareness of potassium restriction after the intervention

### Statistical analysis

(1) Primary endpoint

The mean and standard deviation of serum potassium levels before and after the intervention will be calculated, and a paired student's t-test will be performed at 5% on both sides of the significance level.

(2) Secondary endpoint

The mean and standard deviation of resting blood pressure before and after the intervention will be calculated, and a student's t-test will be performed with a significance level of 5% on both sides. For the content of the questionnaire, Fisher's exact test or chi-square test will be performed at a significance level of 5% on both sides of the change in awareness of potassium limitation.

(3) Comparison of patient backgrounds

Nutritional guidance will be used to compare the patient's background between the improved and non-improving serum potassium groups. For the nominal variables of gender, medical history, medication administration at home, and eating at home, Fisher's exact test or chi-square test is performed at 5% on both sides of the significance level. For the continuous variables age, Body mass index, and eGFR, the mean and standard deviation are calculated, and a student's t-test is performed with a correspondence of 5% on both sides of the significance level.

### Subpopulation analysis

A subpopulation of patients with serum potassium levels greater than or equal to 5.0 mmol/L or less than 5 mmol/L at the time of patient enrollment and low or high awareness of potassium restriction will be generated, and each subpopulation will be analyzed at 3.5.2.

### Interim Analysis

Do not do so.

## Observation items and methods

### Observations and information and samples to be collected

The blood and urine test data used in this study will not be newly tested because the data obtained in daily practice will be obtained retrospectively (retrospectively) through electronic medical records.

(Pre-intervention)

- Patient background: age, gender, body mass index, medical history (heart failure, diabetes), how to manage medications at home (family management or self-management), meals at home (self-catering, home delivery, eating out)
- Blood tests: serum albumin level, Na, Cl, K, Mg, aspartate aminotransferase, alanine aminotransferase, eGFR, blood urea nitrogen, red blood cell count, white blood cell count, Hemoglobin level, hematocrit, platelet count
- Urinalysis: urine albumin, urine albumin/creatinine ratio, or urine protein, urine protein/creatinine ratio
- Oral medications: presence or absence of concomitant medications (mineralocorticoid receptor antagonists, renin-angiotensin-aldosterone inhibitors, sodium glucose cotransporter2 inhibitors) that increase serum potassium levels, presence or absence of concomitant medications (loop diuretics) that reduce serum potassium levels, presence or absence of potassium adsorbents
- Vitals: Resting blood pressure (systolic and diastolic) after visiting the station

In addition, if the blood pressure at home is recorded in the blood pressure notebook, it is possible to substitute the resting blood pressure after the visit.

(12 weeks after intervention)

- Patient background: age, gender, body mass index, medical history (heart failure, diabetes), how to manage medications at home (family management or self-management), meals at home (self-catering, home delivery, eating out)
- Blood tests: serum albumin level, Na, Cl, K, Mg, aspartate aminotransferase, alanine aminotransferase, eGFR, blood urea nitrogen, red blood cell count, white blood cell count, Hemoglobin level, hematocrit, platelet count
- Urinalysis: urine albumin, urine albumin/creatinine ratio, urine protein, urine protein/creatinine ratio
- Oral medications: presence or absence of concomitant medications (mineralocorticoid receptor antagonists, renin-angiotensin-aldosterone inhibitors, sodium glucose cotransporter2 inhibitors) that increase serum potassium levels, presence or absence of concomitant medications (loop diuretics) that reduce serum potassium levels, presence or absence of potassium adsorbents
- Vitals: Resting blood pressure (systolic and diastolic) after visiting the station

In addition, if the blood pressure at home is recorded in the blood pressure notebook, it is possible to substitute the resting blood pressure after the visit.

### Observation and Reporting Schedule

| item | Prescription Fulfillment  (Start of observation) |  |  | End of Observation Period |
| --- | --- | --- | --- | --- |
| Number of days elapsed since obtaining consent | Day1 |  |  | Day 84 onwards* |
| Obtaining Consent | 〇 |  |  |  |
| Questionnaire for awareness survey on the intake of potassium-containing foods | 〇  (Questionnaire 1) |  |  | 〇  (Questionnaire 2) |
| Nutritional Guidance on Potassium Restriction | 〇 |  |  | 〇 |
| Follow-up on Nutrition Guidance |  | 〇 | 〇 |  |
| Patient Background Verification | 〇 |  |  | 〇 |
| Checking blood test data | 〇 |  |  | 〇 |
| Confirmation of urinalysis data | 〇 |  |  | 〇 |
| Confirmation of oral medication | 〇 |  |  | 〇 |
| Measurement of resting blood pressure after the visit | 〇 | 〇 | 〇 | 〇 |
| Confirmation of adherence of potassium adsorbents | 〇 | 〇 | 〇 | 〇 |

*The date of data acquisition after 84 days after obtaining consent depends on the date of the patient's outpatient treatment, so it shall be after Day 84.

### Collection and Evaluation of Adverse Event Information

Once every six months, the principal investigator shall report to the head of the clinical research institute (hospital director) the progress of clinical research and the occurrence of adverse events and defects.

## Discontinuation and termination of the study

### Discontinuation of study subjects

In the following cases, a study subject who has consented to participate in the study may be discontinued from the study:

- When the study subject withdraws consent
- In addition, when the principal investigator deems it

In the event of discontinuation, the research subject will be asked whether or not the data can be used until the discontinuation.

### Discontinuation of the entire study

The Principal Investigator shall consider whether or not to continue the research in the following cases.

1 ) When important information about the quality, safety, or efficacy of the research drug is obtained.

2) When there are no more patients to be observed

3) When it is judged that it is difficult to recruit research subjects and it is extremely difficult to achieve the planned cases.

4) When the Medical Research Ethics Review Committee instructs us to change the implementation plan, etc., and it is judged that it is difficult to accept it.

### Termination of study

The study is terminated when the observation period for all enrolled cases has expired and all data have been fixed, and the analysis of the primary endpoint has been completed.

# Selection Policy for Research Subjects

## Eligibility Criteria

### Criterias of choice

- Disease name and diagnostic method: Patients with an eGFR less than 45 mL/min/1.73 m2
- Stage/type: CKD severity classification stages G3b, G4, G5
- Age: 18 years old or older
- Gender: Regardless
- Indicators of general condition: none
- Associated Major Organ Functions: Renal Function
- Written Patient Consent: Required

### Exclusion Criteria

1. Patients who do not agree to participate in this study
2. Patients who have difficulty communicating
3. Patients with hypokalemia (serum potassium level less than 4.0 mmol/L)
4. Patients undergoing maintenance dialysis
5. Patients Bringing Refill Prescriptions
6. Patients taking potassium preparations internally
7. Patients who are not expected to continue to visit the insurance pharmacy in the study for more than 3 months

## Recruitment of research subjects

Patients with an eGFR of less than 45 ml/min/1.73 m2 who bring an outpatient prescription from Mie Chuo Medical Center between December 1, 2022 ~ February 28, 2023 at the insurance pharmacy of the research institution.

## Registration of study subjects

Registration will be carried out using the case registration form. The principal investigator/co-investigator shall fill in the case registration form with background information such as age and gender of the research subject, and indicate that there are no problems with the inclusion and exclusion criteria. After that, the patient registration form will be sent by fax. By registering, you will be given a case number. The case number, along with information that can identify the study subject, will be transcribed into a correspondence table and kept in a strict security. Do not enter information that can identify an individual, such as a name, in the case registration form.

# Basis for the scientific rationality of the study

It has been reported that the amount of potassium excreted in the urine decreases in CKD stage G3b or later6^)^ and the risk of hyperkalemia is high7^).^ In recent years, it has been shown that pharmacists at community pharmacies improved patient outcomes by providing nutritional guidance for hypertension 8) and diabetes mellitus 9) for a few minutes, in addition to conventional medication guidance. Therefore, it is thought that providing nutritional guidance to CKD patients by pharmacy pharmacists may motivate them to follow a diet at home and contribute to the optimization of serum potassium levels.

# Procedures for obtaining informed consent, etc.

Explanatory documents and consent forms will be prepared by the principal investigator. It will be used after obtaining the approval of the Ethical Review Committee and the permission of the head of the research institution. If you want to revise it, apply to the Ethical Review Committee again, obtain approval, and use it after obtaining permission from the head of the research institution.

In this study, explanations and consent will be obtained by pharmacists at the counter of insurance pharmacies of joint research institutes. Specifically, the director of each joint research institute will explain the outline of the study and the data to be acquired using explanatory documents, and obtain consent from the patient himself/herself. Withdrawal of consent shall be made promptly upon request from the patient and with the signature of the consent withdrawal form.

As a general rule, the matters to be explained to the research subjects when receiving informed consent are as follows. However, this does not apply to matters approved by the head of the research institution in response to the opinion of the Ethical Review Committee.

(1) The name of the research and the fact that permission has been obtained from the head of the research institution to conduct the research.

(2) The name of the research cooperating organization pertaining to the research subject, the name of the person who only provides existing samples and information, the name of the organization to which he belongs, the name of all the principal investigators, and the name of the research institution

(3) Purpose and significance of research

(4) Methods of research (including the purpose of use and handling of samples and information obtained from research subjects) ) and period

(5) Reasons for selection as research subjects

(6) Burden on research subjects and anticipated risks and benefits

(7) The fact that the research can be withdrawn at any time even if the person agrees to the conduct or continuation of the research (if it may be difficult to take measures in accordance with the content of the withdrawal from the research subject, etc., that fact and the reason for the withdrawal will be included). ）

(8) The fact that the research subject will not be treated unfavorably by not agreeing to the conduct or continuation of the research or withdrawing the consent.

(9) Method of disclosure of information on research

(10) At the request of the Research Subject, etc., the fact that the research plan and materials related to the research method can be obtained or viewed to the extent that it does not interfere with the protection of the personal information of other research subjects, etc., and the method of obtaining or viewing such materials

(11) Handling of personal information, etc. (including the method of processing and the creation of pseudonymously processed information or anonymously processed information) ）

Collect information collected in the study from electronic medical records or Google Forms. At that time, information that can identify an individual, such as name and address, is deleted, a code is created to identify the research subject in a way that does not have a regularity with the medical record ID, and a correspondence table between the code and the individual research subject is created. The collected information is information that cannot identify a specific individual unless it is checked against the correspondence table, but it is managed as personal information in this research.

(12) Methods of storage and disposal of samples and information

(13) Conflicts of interest related to research funding sources and other research institutions and conflicts of interest related to individual earnings and other research by researchers, etc.

(14) Handling of research results, etc.

(15) Responding to consultations from research subjects and their related parties (including genetic counseling) ）

(16) If there is an economic burden or gratuity on the research subject, that fact and the details thereof.

(17) In the case of research involving medical treatment that exceeds normal medical care, matters related to other treatment methods, etc.

(18) In the case of research that involves medical treatment beyond normal medical care, measures to be taken regarding the provision of medical care to the research subject after the research is conducted.

(19) In the case of invasive research, whether or not there is compensation for the health damage caused by the research, and the details thereof.

(20) If there is a possibility that the samples and information obtained from the research subject will be used for future research that is not specified at the time of obtaining the consent from the research subject, etc., or that it may be provided to other research institutions, that fact and the content envisaged at the time of obtaining consent.

(21) Invasiveness (excluding minor invasions) In the case of research involving interventions, the person engaged in monitoring, the person engaged in the audit, and the Ethical Review Committee will inspect the samples and information related to the research subject to the extent necessary on the premise that the confidentiality of the research subject will be preserved.

# Handling of Personal Information

Persons involved in research shall comply with applicable laws and ordinances regarding the protection of personal information of research subjects. In addition, the relevant parties shall make every effort to protect the personal information and privacy of the research subjects, and shall not divulge personal information obtained in the course of conducting this research without a justifiable reason. The same shall apply even after the person concerned has retired from the position.

The data will be managed as personal information after changing the name to a research number. Information that can restore personal information (so-called correspondence table) is managed by the personal information manager on a PC that is not connected to the Internet.

# Burdens and anticipated risks and benefits to the study subjects

## Burden and anticipated risks to research subjects

This study is not applicable because it is not invasive.

## Expected benefit to the study subject

Receiving nutritional guidance can be expected to reduce serum potassium levels, resting blood pressure, and urine protein up to 3 months after the intervention compared to before the intervention.

## Comprehensive evaluation of these and measures to minimize burdens and risks

Not applicable.

# Methods of storage and destruction of samples and information

The information obtained in this study will be stored in a locked archive of the Department of Pharmacy, Mie Chuo Medical Center. In addition, insurance pharmacies also store it in a locked library.

As for the disposal method, after the period of time until the date of 5 years from the report of the completion of the study or 3 years from the date of the last publication of the results of the study, whichever is later, the paper media should be shredded and other media should be disposed of in an appropriate manner.

Researchers shall confirm that appropriate procedures are being followed by the person providing the sample or information, and shall prepare a record of the provision of the sample or information. The Principal Investigator shall retain the records prepared by the Researcher for a period of five years from the date of the report of the completion of the study or three years from the date of the last publication of the results of the study, whichever is later. The principal investigator shall keep documents related to the conduct of the research (copies of application documents, notification documents from the hospital director, copies of various applications and reports, lists of research subject identification codes, consent forms, case reports, etc., and other documents or records necessary to guarantee the reliability of the data, etc.), and on the date on which five years have elapsed from the report of the completion of the research. Alternatively, anonymized materials will be discarded after the period of time until the date of the last publication of the research results, whichever is later, has elapsed.

# Contents and methods of reporting to the head of the research institute

The reporting method shall be in accordance with the regulations of each organization.

## Reports from Researchers

Researchers shall report to the head of the research institution in the following cases.

- When serious concerns arise from the viewpoint of respecting the human rights of research subjects, etc., or from the perspective of conducting research, such as leakage of information related to research.
- When facts or information that impairs or may damage the appropriateness of the conduct of research or the credibility of research results are obtained.

## Report from the Principal Investigator

The principal investigator shall report to the head of the research institute in the following cases. In line with this, we will consider suspending or discontinuing the research and changing the research protocol as necessary.

- When facts or information that impairs the ethical validity or scientific rationality of the research, or information that may be impaired and is considered to affect the continuation of the research, is obtained.
- When facts or information that impairs or may damage the appropriateness of the conduct of research or the credibility of research results are obtained.
- Research Progress
- Incidence of adverse events associated with the conduct of the study
- End of study (including in case of discontinuation) If you do. Prepare and report a report on the completion of the study with a summary of the results.

## Report from the Auditor

It is not applicable because it is not audited.

# Conflicts of interest related to research, such as sources of funding for research

There are no companies that fall under the category of conflict of interest. He is currently applying for the Japan Pharmacists Association's Pharmacist Professional Skills Promotion Research Grant Project.

# How to disclose information about research

This study will be registered in jRCT before the start of the study. The results of this research shall be attributed to the principal investigator. The principal investigator, co-investigator, and statistical analyst will discuss and select the authors and report them at academic conferences or papers. Results will not be disclosed to study subjects.

# Responding to consultations from research subjects and related parties

The Research Secretariat will serve as a point of contact for consultations from research subjects and their related parties.

# Procedures for Obtaining Informed Consent from a Substitute, etc.

"Ethical Guidelines for Life Science and Medical Research Involving Human Subjects" Guidance Chapter 4 Informed Consent, etc. Chapter 9 Procedures for Obtaining Informed Consent from Agents, etc. 2 Regarding the "Policy for Selecting Substitutes, etc.," in general, it is basic to select a Representative from among the persons listed in (1) to (3) below.

(1) A person with parental authority or a guardian of a minor (if the research subject is a minor)

(2) Spouses, parents, siblings, children, grandchildren, grandparents, relatives living together, or persons who are considered to be equivalent to their close relatives (excluding minors) ）

(3) A representative of the research subject (including a voluntary guardian who has been granted the right of representation) ）

However, it is desirable that the applicant be selected not uniformly, but rather that a person who is considered to be able to represent the will and interests of the research subject is selected after taking into account the circumstances of each research subject, such as the mental joint relationship such as a partnership or relationship of trust with the research subject, and in some cases the possibility of abuse of the research subject. In addition, when informed consent is received from a substitute, it is also important to keep a record showing the relationship between the substitute and the research subject.

# Procedure for obtaining an informed ascent

The study is for adults 18 years of age and older, subject to the patient's voluntary participation, so ascent is not required.

# Statement of financial burden or gratuity of research subjects, etc., and their details

The present study will be conducted within the normal scope of practice. For this reason, the medical expenses incurred by the study subjects are carried out using the health insurance of the researchers. Participants in this study will not be responsible for transportation expenses or honorariums.

# Response in the event of a serious adverse event

Adverse events that occur within 84 days of the start of the protocol will be followed up until the adverse events improve.

Emergency Reporting

1) The principal investigator/co-investigator will take appropriate measures in the event of a serious adverse event. The co-investigator shall immediately report to the principal investigator, regardless of the causal relationship with the study drug.

2) The Principal Investigator shall immediately report the serious adverse event to the head of the medical institution and notify the Investigator of the Drug Provider. In addition, it will be reported to the Effectiveness and Safety Evaluation Committee as necessary.

3) The report format and procedures for adverse events and adverse events for which a causal relationship with the study drug cannot be ruled out shall be in accordance with the "Procedures for Serious Adverse Events (established by each institution)".

< Emergency Contacts>

Principal Investigator: Yuki Asai

Department of Pharmacy, Mie Chuo Medical Center

〒514-1101 2158-5 Hisai Myojin-cho, Tsu-shi, Mie

Tel: 059-259-1211 FAX: 059-259-0775 (Pharmacy Department)

## Serious adverse events

Adverse events that fall under any of the following are considered "serious adverse events".

1. What leads to death
2. Life-threatening
3. Those that require hospitalization for treatment or extension of the length of hospitalization
4. Permanent or significant disability or malfunction
5. What causes birth defects in offspring

"Unpredictable serious adverse events" means serious adverse events that are not described in the study plan, explanatory documents of informed consent, etc., or that are described but whose nature or severity does not match the description.

## Predictable adverse events

Nutritional guidance on potassium restriction may cause hypokalemia.

## Emergency Reporting

Adverse events that occur within 84 days of the start of the protocol will be followed up until the adverse events improve.

Emergency Reporting

1) The principal investigator/co-investigator will take appropriate measures in the event of a serious adverse event. The co-investigator shall immediately report to the principal investigator, regardless of the causal relationship with the study drug.

2) The Principal Investigator shall immediately report the serious adverse event to the head of the medical institution and notify the Investigator of the Drug Provider. In addition, it will be reported to the Effectiveness and Safety Evaluation Committee as necessary.

3) The report format and procedures for adverse events and adverse events for which a causal relationship with the study drug cannot be ruled out shall be in accordance with the "Procedures for Serious Adverse Events (established by each institution)".

< Emergency Contacts>

Principal Investigator: Yuki Asai

Department of Pharmacy, Mie Chuo Medical Center

〒514-1101 2158-5 Hisai Myojin-cho, Tsu-shi, Mie

Tel: 059-259-1211 FAX: 059-259-0775 (Pharmacy Department)

# Whether or not there is compensation for health damage caused by the research

Although it is not considered that there is a possibility of health damage caused by the participation of the research subject, the principal investigator and the co-investigator will provide appropriate treatment and other necessary measures in the event of a health hazard to the research subject. In this case, the treatment will be covered by insurance, and the research subject will pay the medical expenses for the out-of-pocket expenses.

# Measures to be taken regarding the provision of medical care after the conduct of the research

Treatment after the end of this study is not prescribed.

# Handling of research results related to research subjects

The results of this study will not be disclosed to the research subjects. This is because the study is exploratory and the significance of the results is not significant enough to inform the study subjects because there is currently a lack of certainty.

# Details of outsourcing research-related work and method of supervision of the contractor

Not applicable to this research because it is not outsourced.

# If there is a possibility that the samples and information obtained from the research subject will be used for future research that is not specified at the time of obtaining the consent from the research subject, etc., or that may be provided to other research institutions, that fact and the contents that are expected at the time of obtaining the consent

In order to check the accuracy of the data, the publisher of the paper may ask for the data, or it may be provided to researchers around the world to use the data (data sharing). In this case, the data will be processed so that individuals cannot be identified, and a correspondence table will not be provided.

# When monitoring and auditing are to be conducted, the implementation system and procedures

## monitoring

The study is not invasive, so no monitoring will be performed.

## audit

The study was not invasive, so no audits were conducted.

# other

## references

1. Ministry of Health, Labour and Welfare Kidney Disease Control Study Group Report
2. Palmer BF, Carrero JJ, Clegg DJ, Colbert GB, Emmett M, Fishbane S, Hain DJ, Lerma E, Onuigbo M, Rastogi A, Roger SD, Spinowitz BS, Weir MR. Clinical Management of Hyperkalemia. Mayo Clin Proc. 2021 Mar; 96(3):744-762.
3. Stevens PE, Levin A; Kidney Disease: Improving Global Outcomes Chronic Kidney Disease Guideline Development Work Group Members. Evaluation and management of chronic kidney disease: synopsis of the kidney disease: improving global outcomes 2012 clinical practice guideline. Ann Intern Med. 2013 Jun 4; 158(11):825-830
4. Morris A, Krishnan N, Kimani PK, Lycett D. Effect of Dietary Potassium Restriction on Serum Potassium, Disease Progression, and Mortality in Chronic Kidney Disease: A Systematic Review and Meta-Analysis. J Ren Nutr. 2020 Jul; 30(4):276-285.
5. Kurita N, Wakita T, Ishibashi Y, Fujimoto S, Yazawa M, Suzuki T, Koitabashi K, Yanagi M, Kawarazaki H, Green J, Fukuhara S, Shibagaki Y. Association between health-related hope and adherence to prescribed treatment in CKD patients: multicenter cross-sectional study. BMC Nephrol. 2020 Oct 31; 21(1):453.
6. Ueda Y, Ookawara S, Ito K, Miyazawa H, Kaku Y, Hoshino T, Tabei K, Morishita Y. Changes in urinary potassium excretion in patients with chronic kidney disease. Kidney Res Clin Pract. 2016 Jun; 35(2):78-83.
7. Saito Y, Yamamoto H, Nakajima H, Takahashi O, Komatsu Y. Incidence of and risk factors for newly diagnosed hyperkalemia after hospital discharge in non-dialysis-dependent CKD patients treated with RAS inhibitors. PLoS One. 2017 Sep 6; 12(9):e0184402.
8. Okada H, Onda M, Shoji M, Sakane N, Nakagawa Y, Sozu T, Kitajima Y, Tsuyuki RT, Nakayama T. Effects of lifestyle advice provided by pharmacists on blood pressure: The COMmunity Pharmacists ASSist for Blood Pressure (COMPASS-BP) randomized trial. Biosci Trends. 2018 Jan 9; 11(6):632-639.
9. Hiroshi Okada, Mitsuko Onda, Masaki Shoji, Kazuhiko Kotani, Takeo Nakayama, Yasushi Nakagawa, Naoki Sakane. Effects of Lifestyle Intervention Performed by Community Pharmacists on Glycemic Control in Patients with Type 2 Diabetes: The Community Pharmacists Assist (Compass) Project, a Pragmatic Cluster Randomized Trial. Pharmacology & Pharmacy. 2016 Mar,7(3):124-132.

## Appendix

1. Dietary guidance: Explanatory documents on nutritional guidance for CKD

2. Questionnaire of the Awareness Survey on the Intake of Potassium-Containing Foods (Before the Intervention) (Questionnaire 1)

3. Questionnaire of the Awareness Survey on the Intake of Potassium-Containing Foods (Post-Intervention) (Questionnaire 2)
