# Supplementary material for: A multicentral prospective cohort trial of a pharmacist-led nutritional intervention on serum potassium levels in outpatients with chronic kidney disease: The MieYaku-Chronic Kidney Disease project
Source: PLoS One. 2024 May 31;19(5):e0304479. doi: 10.1371/journal.pone.0304479 (PMC11142692; doi:10.1371/journal.pone.0304479)
Supplement: S2 File — (DOCX) [file pone.0304479.s004.docx]

**研究実施計画書**

**慢性腎臓病患者における血清カリウム値の適正化を目指した**

**地域薬局の介入効果の検討**

　　略称：　Mieyaku-chronic kidney disease: My-CKD試験

研究代表者：国立病院機構三重中央医療センター　薬剤部

　　　　　　　　　　　　　　　　　　　　　　　　　　　　　　　　　　　　　　　　　　　　　　　　　　　　朝居　祐貴

研究事務局：国立病院機構三重中央医療センター　薬剤部

　　　　　　　　朝居　祐貴

2022年11月4日　作成　　Ver 1.0

2022年11月24日　作成　Ver 2.0

【作成・改訂履歴】

| 版番号 | 作成日／改訂日 | 改訂理由 |
| --- | --- | --- |
| Ver.1.0 | 2022年11月4日 | 新規作成 |
| Ver. 2.0 | 2022年11月24日 | 倫理審査委員会終了後の改訂 |
|  |  |  |
|  |  |  |
|  |  |  |

**目次**

[0. シェーマ 1](#_Toc120209233)

[0.1. 研究デザインの要約 2](#_Toc120209234)

[1. 研究の実施体制 3](#_Toc120209235)

[1.1. 研究代表責任者 3](#_Toc120209236)

[1.2. 研究事務局 3](#_Toc120209237)

[1.3. 研究責任者 3](#_Toc120209238)

[1.4. 研究分担者 4](#_Toc120209239)

[1.5. 統計解析責任者 4](#_Toc120209240)

[1.6. データマネジメント責任者 4](#_Toc120209241)

[1.7. 個人情報管理者 4](#_Toc120209242)

[1.8. モニタリング責任者 4](#_Toc120209243)

[1.9. 監査責任者 4](#_Toc120209244)

[2. 研究の目的及び意義 4](#_Toc120209245)

[2.1. 目的 4](#_Toc120209246)

[2.1.1. 主要目的 4](#_Toc120209247)

[2.1.2. 副次目的 5](#_Toc120209248)

[2.2. 背景 5](#_Toc120209249)

[2.3. 研究の意義 5](#_Toc120209250)

[3. 研究の方法及び期間 5](#_Toc120209251)

[3.1. 研究デザイン（研究方法） 5](#_Toc120209252)

[3.2. 予定研究対象者数及び設定根拠 7](#_Toc120209253)

[3.2.1. 予定研究対象者数 7](#_Toc120209254)

[3.2.2. 予定研究対象者数の設定根拠 7](#_Toc120209255)

[3.3. 研究期間 7](#_Toc120209256)

[3.4. データ収集 7](#_Toc120209257)

[様式と提出期限 7](#_Toc120209258)

[記入方法 7](#_Toc120209259)

[送付方法 8](#_Toc120209260)

[3.5. 統計解析の方法 8](#_Toc120209261)

[3.5.1. 解析対象集団 8](#_Toc120209262)

[3.5.2. 統計解析 8](#_Toc120209263)

[3.5.3. 部分集団解析 8](#_Toc120209264)

[3.5.4. 中間解析 9](#_Toc120209265)

[3.6. 観察項目及び方法 9](#_Toc120209266)

[3.6.1. 観察項目と収集する情報および試料 9](#_Toc120209267)

[3.6.2. 観察・報告スケジュール 10](#_Toc120209268)

[3.6.3. 有害事象情報の収集と評価について 10](#_Toc120209269)

[3.7. 研究の中止と終了 10](#_Toc120209270)

[3.7.1. 研究対象者の中止 10](#_Toc120209271)

[3.7.2. 研究全体の中止 10](#_Toc120209272)

[3.7.3. 研究の終了 11](#_Toc120209273)

[4. 研究対象者の選定方針 11](#_Toc120209274)

[4.1. 適格基準 11](#_Toc120209275)

[4.1.1. 選択基準 11](#_Toc120209276)

[4.1.2. 除外基準 11](#_Toc120209277)

[4.2. 研究対象者のリクルート 11](#_Toc120209278)

[4.3. 研究対象者の登録 11](#_Toc120209279)

[5. 研究の科学的合理性の根拠 12](#_Toc120209280)

[6. インフォームド・コンセントを受ける手続き等 12](#_Toc120209281)

[7. 個人情報の取扱い 14](#_Toc120209282)

[8. 研究対象者に生じる負担並びに予測されるリスク及び利益 14](#_Toc120209283)

[8.1. 研究対象者に生じる負担並びに予測されるリスク 14](#_Toc120209284)

[8.2. 研究対象者に予想される利益 14](#_Toc120209285)

[8.3. これらの総合評価並びに負担及びリスクを最小化する対策 14](#_Toc120209286)

[9. 試料・情報の保管及び破棄の方法 14](#_Toc120209287)

[10. 研究機関の長への報告内容及び方法 15](#_Toc120209288)

[10.1. 研究者等からの報告 15](#_Toc120209289)

[10.2. 研究責任者からの報告 15](#_Toc120209290)

[10.3. 監査担当者からの報告 15](#_Toc120209291)

[11. 研究の資金源等研究に係る利益相反 15](#_Toc120209292)

[12. 研究に関する情報公開の方法 15](#_Toc120209293)

[13. 研究対象者等及びその関係者からの相談等への対応 16](#_Toc120209294)

[14. 代諾者等からインフォームド・コンセントを受ける場合の手続き 16](#_Toc120209295)

[15. インフォームド・アセントを得る場合の手続き 16](#_Toc120209296)

[16. 研究対象者等の経済的負担又は謝礼の旨及びその内容 16](#_Toc120209297)

[17. 重篤な有害事象が発生した際の対応 16](#_Toc120209298)

[17.1. 重篤な有害事象 17](#_Toc120209299)

[17.2. 予測できる有害事象 17](#_Toc120209300)

[17.3. 緊急報告 17](#_Toc120209301)

[18. 研究によって生じた健康被害に対する補償の有無 18](#_Toc120209302)

[19. 研究実施後における医療の提供に関する対応 18](#_Toc120209303)

[20. 研究対象者に係る研究結果の取扱い 18](#_Toc120209304)

[21. 研究に関する業務を委託する場合の内容及び委託先の監督方法 18](#_Toc120209305)

[22. 研究対象者から取得された試料・情報について研究対象者等から同意を受ける時点では特定されない将来の研究のために用いられる可能性又は他の研究機関に提供する可能性がある場合には、その旨と同意を受ける時点において想定される内容 18](#_Toc120209306)

[23. モニタリング及び監査を実施する場合には、その実施体制及び実施手順 18](#_Toc120209307)

[23.1. モニタリング 18](#_Toc120209308)

[23.2. 監査 19](#_Toc120209309)

[24. その他 19](#_Toc120209310)

[24.1. 文献 19](#_Toc120209311)

[24.2. 別添 20](#_Toc120209312)

# シェーマ

- 主な適格規準：国立病院機構三重中央医療センターに通院する推定糸球体ろ過量（以下、eGFR）45 mL/min/1.73 m^2^未満の患者
- 患者登録のタイミング：登録期間にて研究協力施設の保険薬局に来局した初回日
- 症例数および登録期間：40名、2022年12月～2023年2月28日
- 介入方法：

1．カリウム含有食品の摂取についての意識調査のアンケート調査

2．食事療法の指導：食事療法に関する説明文書を用いて栄養指導

3. 来局後の安静時血圧の測定

4．カリウム吸着薬の服薬状況について三重中央医療センターへの情報共有

- 追跡期間：介入初日より3カ月後(84日後)

研究デザイン

- 前向き
- 介入研究
- デザインの特徴：前後比較試験、オープンデザイン
- 対照の種類：なし
- 実施施設：多施設
- ランダム化：無
- 盲検化のレベル：非盲検


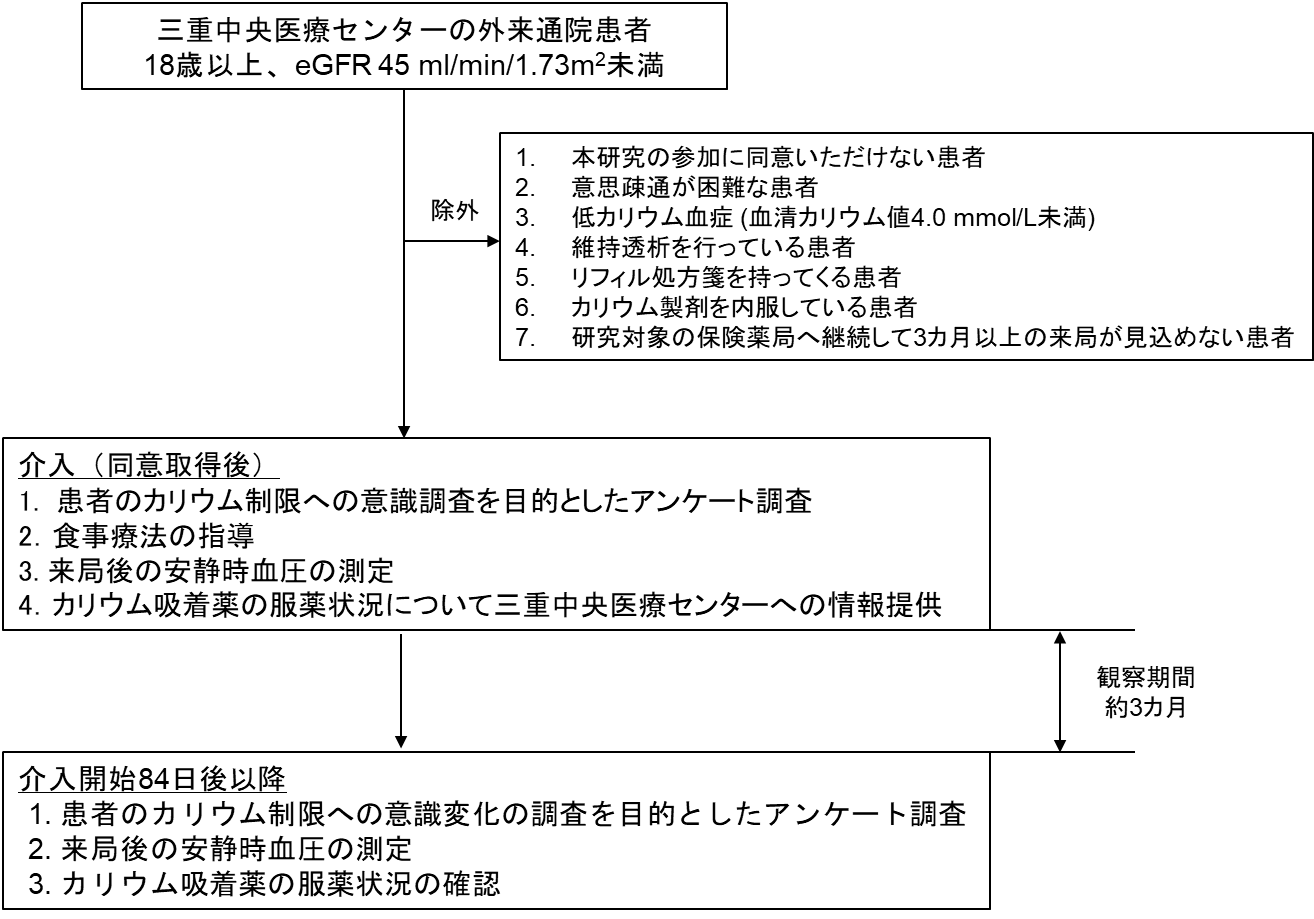


## 研究デザインの要約

前向き介入、前後比較、オープンデザイン

本研究は、『個人情報保護法』、『ヘルシンキ宣言』及び『人を対象とする生命科学・医学系研究に関する倫理指針』を遵守して行う。

# 研究の実施体制

## 研究代表責任者

三重中央医療センター　　　薬剤部　　　 薬剤師　　　 朝居祐貴

## 研究事務局

三重中央医療センター　　　薬剤部　　　 薬剤師　　　 朝居祐貴

（連絡先）

住 所　： 〒514-1101　津市久居明神町2158-5

電 話　： 059-259-1211 （内線4724）

FAX　　： 059-256-2651

E-mail ： yuki0715asai@gmail.com

## 研究責任者

全23施設の保険薬局

スギ薬局久居インターガーデン店 　薬剤師　宮田香織

薬局アイファーマシィー明神店　薬剤師　伊豆川　愛

久居調剤薬局　薬剤師　小林　竜也

スギ薬局久居新町店　薬剤師　長谷部　智春

こころ調剤薬局　薬剤師　乾　浩也

こうなん薬局　薬剤師　村阪 敏規

久居センター薬局　薬剤師　高崎 郁宏

なの花薬局観音寺店　薬剤師　末澤千恵

つばめ薬局　薬剤師　内藤　潤

ペンギン薬局　薬剤師　佐藤亮

一志調剤薬局・高野店　薬剤師　中川裕司

ドレミ薬局　薬剤師　新美　有子

久居野村調剤薬局　薬剤師　荻野　恭希

健やか薬局明神店　薬剤師　杉野宏樹

久居新町薬局　薬剤師　小林亮太

高茶屋薬局　薬剤師　木下仁郎

スギ薬局津新町店　薬剤師　福山孝宏

メディモ調剤薬局　薬剤師　寺田幸司

フラワー薬局一志店　薬剤師　裏川　祐加

ココカラファイン薬局垂水店　薬剤師　宮地浩典

太陽薬局　薬剤師　青木　智彦

明薬局　薬剤師　松室 泰輔

一志調剤薬局・中町店　薬剤師　中川信之

## 研究分担者

三重中央医療センター 　　薬剤部　　　 薬剤師　　 　 簗川　樹

三重中央医療センター　　 薬剤部　　 　 薬剤部長　 佐藤賛治

三重中央医療センター　　 栄養科　　　 管理栄養士　 村松麻美

三重中央医療センター　　 循環器内科　 医師　　 　岡崎貴大

## 統計解析責任者

三重中央医療センター　　　薬剤部　　　 薬剤師　　　 朝居祐貴

## データマネジメント責任者

設置しない。

## 個人情報管理者

三重中央医療センター　　　薬剤部　　　 薬剤師　　　 朝居祐貴

## モニタリング責任者

該当なし。

## 監査責任者

該当なし。

# 研究の目的及び意義

## 目的

慢性腎臓病 (以下、CKD) 患者を対象に従来の服薬指導に加えて、薬局薬剤師による数分間のカリウム制限に関する栄養指導が血清カリウム値へ及ぼす影響を明らかにする。

主要評価項目は血清カリウム値、副次評価項目は来局後の安静時血圧、患者のカリウム制限に対する意識の変化とする。

### 主要目的

①介入前後における血清カリウム値の変化

②介入前後におけるカリウム含有食品の摂取についての意識の変化

### 副次目的

| 副次目的 | 評価項目 | 評価項目の選択理由と妥当性 |
| --- | --- | --- |
| カリウム含有食品の摂取についての意識変化 | アンケート調査結果 | カリウム制限への意識に関する設問に対して5段階評価にて評価 |
| 介入前後での安静時血圧の変化 | 来局時の安静時での収縮期血圧と拡張期血圧 | 食事療法により血圧の変動が期待される |

## 背景

腎機能が高度に低下したCKD患者は2016年時点で約33万人と報告されており、今後もさらなる増加が予測されている^1)^。CKD患者は、腎機能の低下に伴い様々な有害事象を併発するが、特に高カリウム血症は最も頻度が高い電解質異常であり、不整脈や突然死を引き起こすことがある。

高カリウム血症は、食事由来の過剰なカリウム摂取が引き金となるため^2)^、多くのCKD患者はカリウム摂取制限への指導が重要である。しかしながら、入院加療中では食事や薬物療法が厳格にコントロールされているために適正な血清カリウム値を示しているが、退院後では食事療法やカリウム吸着薬のアドヒアランスを維持することができないため、高カリウム血症にて緊急入院される症例が散見される。

## 研究の意義

地域密着型医療に貢献している保険薬局が、カリウム制限への栄養指導を実施することで、患者の自宅での食事療法維持への動機付けとなり、高カリウム血症による緊急入院を予防できる可能性がある。高カリウム血症は、致死的な有害事象のため、患者の生命予後を改善する可能性がある。

# 研究の方法及び期間

## 研究デザイン（研究方法）

・症例登録

CKD重症度分類のステージG3b、G4、G5^3)^

三重中央医療センターの外来院外処方箋を保険薬局へ持参した際に、患者本人に文書による同意を取り、登録する。同意取得日をDay1とする。

・介入方法

以下の介入はすべて保険薬局の薬剤師が実施する。

同意取得日（Day1）

1．患者のカリウム含有食品の摂取についての意識調査のアンケート（アンケート1）別添2

2．食事療法の指導：CKDに対する栄養指導に関する説明文書（別添1）を用いて指導する。(必要に応じて栄養管理室への栄養指導の勧奨)

3. 来局後の安静時血圧の測定

4．カリウム吸着薬の服薬状況について三重中央医療センターへの情報提供：アドヒアランス不良の原因が剤型や用法の場合、処方元の三重中央医療センターへトレーシングレポートにて情報提供を行う。

⇒病院薬剤師が処方医へ電子カルテの掲示板に服用状況などの詳細を記載し、剤型変更を提案する。

Day1-83

1. 食事療法の指導（必要に応じて適宜実施）

2. 来局後の安静時血圧の測定

3. カリウム吸着薬の服薬状況の確認（三重中央医療センターへ情報提供を行った場合、剤型変更等の確認）

Day84以降（観察期間終了日）

1．患者のカリウム含有食品の摂取についての意識調査のアンケート（アンケート2） 別添3

2. 来局後の安静時血圧の測定

3. カリウム吸着薬の服薬状況の確認（三重中央医療センターへ情報提供を行った場合、剤型変更等の確認）

アンケート内容

すべての設問において、5段階の回答を設定する。

同意取得時 (Day1)：アンケート1

Q1. 血液中のカリウムが高すぎるといけないことを知っていますか？

Q2. カリウムの含まれる量が多い食品を知っていますか？

Q3. カリウムの含まれる量が多い食品を控えるなど、普段から意識していますか？

Q4. カリウム摂取量を意識するのは、煩わしい（面倒くさい）ですか？

同意取得3カ月後 (Day84以降) ：アンケート2

Q1. 血液中のカリウムが高すぎるといけないことを知っていますか？

Q2. カリウムの含まれる量が多い食品を知っていますか？

Q3. カリウムの含まれる量が多い食品を控えるなど、普段から意識していますか？

Q4. カリウム摂取量を意識するのは、煩わしい（面倒くさい）ですか？

Q5. 今回の指導によりカリウムの含まれる食品の摂取に気を付けるようになりましたか？

Q6. 今後もカリウムの含まれる食品への対策を続けていきたいと思いますか？

Q7. なにか今回の指導を通じてお気づきの点がありましたら教えてください。

・観察期間

同意取得日(Day1)より計算し、12週（Day84）以降とする

・データ取得

観察期間中に保険薬局へ来局する度に、安静時血圧（収縮期血圧、拡張期血圧）を測定し、記録する。本研究は非ランダム化で行う。

## 予定研究対象者数及び設定根拠

### 予定研究対象者数

共同研究施設全体で40例

### 予定研究対象者数の設定根拠

既報では、カリウム制限食への栄養指導により、最大0.5 mmol/Lの血清カリウム値の低下が認められている^4)^。一般的に、血清カリウム値の個人間における標準偏差は0.5 mmol/L程度であったことが報告されている^5)^。しかし、Kuritaらの報告^5)^はn=60-70での標準偏差であり、本研究での標準偏差は既報より大きくなる可能性がある。そのため、本研究での標準偏差は1.0 mmol/Lと見積もった。対応のあるt検定にてα=0.8、β=0.05、母集団の血清カリウム値の標準偏差が1.0 mmol/L、介入による平均値の差を0.5 mmol/Lとすると、必要症例数はn=34と算出される。脱落者も加味してn=40を目標とした。

三重中央医療センターでの2022年5月1日から7月31日の3カ月間における該当患者（eGFR<45 mL/min/1.73m^2^かつ血清カリウム値が4.0 mmol/L以上）は1344名であり、多くが共同研究機関の保険薬局へ来局していることから、実施可能であると考える。

## 研究期間

登録期間　許可日（またはjRCT公表日）から2023年2月28日

　　　研究期間　許可日（またはjRCT公表日）から2023年5月31日

　　　参加期間　6か月

## データ収集

## 様式と提出期限

本研究では、介入前後におけるアンケート結果と、Googleフォームを用いた以下の項目について、研究代表者へ提出する。なお、Googleフォームを用いた以下の項目の提出期限は患者の観察期間が終了した1週間以内とする。

## 記入方法

Googleフォーム内にて、薬局名、研究登録番号、介入日、自宅での薬の管理方法（本人管理・家族管理・施設管理・介護士管理）、直近の自宅での食事について（自炊、宅配食、外食）、栄養士による詳細な栄養指導の希望の有無は選択式、来局時の安静時血圧は記述式を用いる。

なお、研究登録番号は、患者から本研究への参加の同意後、研究代表者より各施設の代表者へ研究登録番号を伝達する。研究登録番号と患者ID、患者名は対応表を作成し、研究責任者が保管する。

## 送付方法

Googleフォーム内の項目は、Web上での提出様式を用いる。アンケート結果についてはFAXを用いる。

## 統計解析の方法

### 解析対象集団

主要エンドポイントおよび副次エンドポイントの解析は、Full Analysis Setを対象としたものを主解析とする。また、Per Protocol Setを対象とした解析も実施し、解析結果の安定性を確認する。

①Full Analysis Setの定義

　　以下の基準を全て満たす症例とする。

　　（1）三重中央医療センターに外来通院したeGFR　45 ml/min/1.73 m^2^未満および血清カリウム値が4.0 mmol/L以上

　　（2）保険薬局にて本研究の同意を取得した患者

②Per Protocol Setの定義

　　FASのうち、以下の基準を全て満たす症例とする。

　　（1）同意取得後84日以降でのアンケート2の結果が得られた患者

　　（2）同意取得後84日以降での来局後の安静時血圧が得られた患者

　　（3）介入後でのカリウム制限への意識に関するアンケートの回答が得られた患者

### 統計解析

①主要エンドポイント

血清カリウム値について、介入前と介入後での平均値と標準偏差を算出するとともに、有意水準両側5%で対応のあるStudent’s t-testを行う。

②副次エンドポイント

安静時血圧について、介入前と介入後での平均値と標準偏差を算出するとともに、有意水準両側5%で対応のあるStudent’s t-testを行う。アンケート内容については、カリウム制限に対する意識の変化を有意水準両側5%でFisherの正確確率検定またはカイ二乗検定を実施する。

③患者背景の比較

栄養指導により、血清カリウム値の改善群と非改善群での患者背景の比較を行う。名義変数である性別、既往歴、自宅での薬の管理方法、自宅での食事については、有意水準両側5%でFisherの正確確率検定またはカイ二乗検定を実施する。連続変数である年齢、Body mass index、eGFRについては、平均値と標準偏差を算出するとともに、有意水準両側5%で対応のあるStudent’s t-testを行う。

### 部分集団解析

患者登録時の血清カリウム値が5.0 mmol/L以上または5 mmol/L未満、カリウム制限への意識が低い患者または高い患者にて部分集団を作成して、それぞれの部分集団で3.5.2の解析を行う。

### 中間解析

実施しない。

## 観察項目及び方法

### 観察項目と収集する情報および試料

本研究で使用する血液検査および尿検査データは、日常診療で得られたデータを後方視的（後ろ向き）に電子カルテにより入手するため、新たに検査を実施することはない。

（介入前）

- 患者背景：年齢、性別、Body mass index、既往歴（心不全、糖尿病）、自宅での薬の管理方法（家族管理か自己管理）、自宅での食事について（自炊、宅配食、外食）
- 血液検査：血清アルブミン値、Na、Cl、K、Mg、アスパラギン酸アミノトランスフェラーゼ、アラニンアミノトランスフェラーゼ、eGFR、血中尿素窒素、赤血球数、白血球数、ヘモグロビン値、ヘマトクリット値、血小板数
- 尿検査：尿アルブミン、尿アルブミン/クレアチニン比、または尿たんぱく、尿たんぱく/クレアチニン比
- 内服薬：血清カリウム値を上昇させる併用薬（ミネラルコルチコイド受容体拮抗薬、レニンアンギオテンシン-アルドステロン阻害薬、sodium glucose cotransporter2 阻害薬）の有無、血清カリウム値を低下させる併用薬（ループ利尿薬）の有無、カリウム吸着薬の有無
- バイタル：来局後の安静時血圧（収縮期・拡張期）

なお、血圧手帳にて自宅での血圧を記録している場合、来局後の安静時血圧の代替可能とする。

（介入12週後）

- 患者背景：年齢、性別、Body mass index、既往歴（心不全、糖尿病）、自宅での薬の管理方法（家族管理か自己管理）、自宅での食事について（自炊、宅配食、外食）
- 血液検査：血清アルブミン値、Na、Cl、K、Mg、アスパラギン酸アミノトランスフェラーゼ、アラニンアミノトランスフェラーゼ、eGFR、血中尿素窒素、赤血球数、白血球数、ヘモグロビン値、ヘマトクリット値、血小板数
- 尿検査：尿アルブミン、尿アルブミン/クレアチニン比、尿たんぱく、尿たんぱく/クレアチニン比
- 内服薬：血清カリウム値を上昇させる併用薬（ミネラルコルチコイド受容体拮抗薬、レニンアンギオテンシン-アルドステロン阻害薬、sodium glucose cotransporter2 阻害薬）の有無、血清カリウム値を低下させる併用薬（ループ利尿薬）の有無、カリウム吸着薬の有無
- バイタル：来局後の安静時血圧（収縮期・拡張期）

なお、血圧手帳にて自宅での血圧を記録している場合、来局後の安静時血圧の代替可能とする。

### 観察・報告スケジュール

| 項目 | 処方箋の応需  （観察開始） |  |  | 観察期間の終了 |
| --- | --- | --- | --- | --- |
| 同意取得後経過日数 | Day1 |  |  | Day84以降* |
| 同意取得 | 〇 |  |  |  |
| カリウム含有食品の摂取についての意識調査のアンケート | 〇  (アンケート1) |  |  | 〇  (アンケート2) |
| カリウム制限に関する栄養指導 | 〇 |  |  | 〇 |
| 栄養指導のフォロー |  | 〇 | 〇 |  |
| 患者背景の確認 | 〇 |  |  | 〇 |
| 血液検査データの確認 | 〇 |  |  | 〇 |
| 尿検査データの確認 | 〇 |  |  | 〇 |
| 内服薬の確認 | 〇 |  |  | 〇 |
| 来局後の安静時血圧の測定 | 〇 | 〇 | 〇 | 〇 |
| カリウム吸着薬のアドヒアランスの確認 | 〇 | 〇 | 〇 | 〇 |

*同意取得後84日以降のデータ取得日は、患者の外来診療日に依存するため、Day84以降とする。

### 有害事象情報の収集と評価について

研究責任者は半年に1回、臨床研究の進捗状況ならびに有害事象および不具合等の発生状況等を臨床研究機関の長（病院長）に報告する。

## 研究の中止と終了

### 研究対象者の中止

次のような場合、研究参加の同意を取得した研究対象者の研究参加を中止する可能性がある。

- 研究対象者が同意を撤回した場合
- その他に研究責任者が判断した場合

中止の際は、中止までのデータの使用可否について研究対象者に確認を行う。

### 研究全体の中止

研究責任者は、以下の事項に該当する場合は研究実施継続の可否を検討する。

1 ) 研究薬の品質、安全性、有効性に関する重大な情報が得られたとき

2) 観察対象患者がいなくなったとき

3) 研究対象者のリクルートが困難で予定症例を達成することが到底困難であると判断されたとき

4)　医学系研究倫理審査委員会により、実施計画等の変更の指示があり、これを受入れることが困難と判断されたとき

### 研究の終了

全ての登録症例における観察期間が満了し、全てのデータが固定された後、主要エンドポイントの解析が終了した時点で研究終了とする。

# 研究対象者の選定方針

## 適格基準

### 選択基準

- 疾患名および診断方法：eGFR 45 mL/min/1.73 m^2^未満の患者
- 病期・病型：CKD重症度分類のステージG3b、G4、G5
- 年齢：18歳以上
- 性別：問わない
- 全身状態の指標:なし
- 関連する主要臓器機能：腎機能
- 文書による患者の同意：必要

### 除外基準

1. 本研究の参加に同意いただけない患者
2. 意思疎通が困難な患者
3. 低カリウム血症の患者 (血清カリウム値4.0 mmol/L未満)
4. 維持透析を行っている患者
5. リフィル処方箋を持ってくる患者
6. カリウム製剤を内服している患者
7. 研究対象の保険薬局へ継続して3カ月以上の来局が見込めない患者

## 研究対象者のリクルート

研究実施機関の保険薬局にて、2022年12月1日～2023年2月28日の間に三重中央医療センターの外来処方箋を持ってくるeGFR 45 ml/min/1.73 m^2^未満の患者。

## 研究対象者の登録

登録は症例登録票を用いて行う。研究責任者/分担者は症例登録票に研究対象者の年齢、性別等の背景情報を記入し、選択基準・除外基準に問題がないことを記入する。その後、症例登録票をFAXにて送信する。登録することで、症例番号が付与される。症例番号は、研究対象者を識別できる情報と共に対応表に転記し、厳重に保管する。症例登録票には名前などの個人を識別できる情報は入力しない。

# 研究の科学的合理性の根拠

CKDステージ分類G3b以降では尿中へのカリウム排泄量が低下し^6)^、高カリウム血症のリスクが高いことが報告されている^7)^。近年、地域薬局の薬剤師が従来の服薬指導に加えて、数分間の高血圧^8)^や糖尿病^9)^に対する栄養指導が患者のアウトカムを改善させたことが示されている。従って、薬局薬剤師のCKD患者に対する栄養指導を行うことが自宅での食事療法への動機付けとなり、血清カリウム値の適正化に貢献できる可能性が考えられる。

# インフォームド・コンセントを受ける手続き等

説明文書および同意書は研究責任者が作成する。倫理審査委員会の承認及び研究機関の長の許可を得た後に、使用する。改訂する場合は再度倫理審査委員会に申請し、承認を得た後、研究機関の長の許可を受けた後に使用する。

本研究では、説明および同意取得は、共同研究機関の保険薬局の窓口にて薬剤師が行う。具体的な手順としては、各共同研究機関の責任者が説明文書を用いて、本研究の概要・取得するデータについて説明し、患者本人より同意を取得する。同意撤回は、患者本人より申し出があった際に、速やかに同意撤回書への署名のもと行う。

インフォームド・コンセントを受ける際に研究対象者等に対し説明すべき事項は、原則として以下のとおりとする。ただし、倫理審査委員会の意見を受けて研究機関の長が許可した事項については、この限りでない。

① 研究の名称及び当該研究の実施について研究機関の長の許可を受けている旨

② 当該研究対象者に係る研究協力機関の名称、既存試料・情報の提供のみを行う者の氏名及び所属する機関の名称並びに全ての研究責任者の氏名及び研究機関の名称

③ 研究の目的及び意義

④ 研究の方法（研究対象者から取得された試料・情報の利用目的及び取扱いを含む。）及び期間

⑤ 研究対象者として選定された理由

⑥ 研究対象者に生じる負担並びに予測されるリスク及び利益

⑦ 研究が実施又は継続されることに同意した場合であっても随時これを撤回できる旨（研究対象者等からの撤回の内容に従った措置を講ずることが困難となる場合があるときは、その旨及びその理由を含む。）

⑧ 研究が実施又は継続されることに同意しないこと又は同意を撤回することによって研究対象者等が不利益な取扱いを受けない旨

⑨ 研究に関する情報公開の方法

⑩ 研究対象者等の求めに応じて、他の研究対象者等の個人情報等の保護及び当該研究の独創性の確保に支障がない範囲内で研究計画書及び研究の方法に関する資料を入手又は閲覧できる旨並びにその入手又は閲覧の方法

⑪ 個人情報等の取扱い（加工する場合にはその方法、仮名加工情報又は匿名加工情報を作成する場合にはその旨を含む。）

研究で収集する情報を電子カルテまたはGoogleフォームから収集する。その際、名前や住所などの個人を識別できる情報は削除し、カルテIDとの規則性を有さない方法で研究対象者を識別するコードを作成し、当該コードと研究対象者個人との対応表を作成する。収集した情報は対応表と照合しない限り特定の個人が識別できない情報であるが、本研究では個人情報として管理する。

⑫ 試料・情報の保管及び廃棄の方法

⑬ 研究の資金源その他の研究機関の研究に係る利益相反、及び個人の収益その他の研究者等の研究に係る利益相反に関する状況

⑭ 研究により得られた結果等の取扱い

⑮ 研究対象者等及びその関係者からの相談等への対応（遺伝カウンセリングを含む。）

⑯ 研究対象者等に経済的負担又は謝礼がある場合には、その旨及びその内容

⑰ 通常の診療を超える医療行為を伴う研究の場合には、他の治療方法等に関する事項

⑱ 通常の診療を超える医療行為を伴う研究の場合には、研究対象者への研究実施後における医療の提供に関する対応

⑲ 侵襲を伴う研究の場合には、当該研究によって生じた健康被害に対する補償の有無及びその内容

⑳ 研究対象者から取得された試料・情報について、研究対象者等から同意を受ける時点では特定されない将来の研究のために用いられる可能性又は他の研究機関に提供する可能性がある場合には、その旨と同意を受ける時点において想定される内容

㉑ 侵襲（軽微な侵襲を除く。）を伴う研究であって介入を行うものの場合には、研究対象者の秘密が保全されることを前提として、モニタリングに従事する者及び監査に従事する者並びに倫理審査委員会が、必要な範囲内において当該研究対象者に関する試料・情報を閲覧する旨

# 個人情報の取扱い

研究に関わる関係者は、研究対象者の個人情報保護について、適用される法令、条例を遵守する。 また関係者は、研究対象者の個人情報およびプライバシー保護に最大限の努力を払い、本研究を行う上で知り得た個人情報を正当な理由なく漏らしてはいけない。関係者がその職を退いた後も同様とする。

データは、氏名を研究用番号に変更したうえで、個人情報として管理する。個人情報を復元できる情報（いわゆる対応表）はネットのつながっていないPCで個人情報管理者が管理する。

# 研究対象者に生じる負担並びに予測されるリスク及び利益

## 研究対象者に生じる負担並びに予測されるリスク

本研究は、侵襲性はないため該当しない。

## 研究対象者に予想される利益

栄養指導を受けることで、介入前と比較して介入3か月後までの血清カリウム値、安静時血圧や尿たんぱくの低下が期待できる。

## これらの総合評価並びに負担及びリスクを最小化する対策

該当なし。

# 試料・情報の保管及び破棄の方法

本研究で得られた情報は三重中央医療センター薬剤部の鍵付き書庫に保管する。なお、保険薬局においても鍵付き書庫に保管する。

廃棄方法については、研究の終了の報告から5年を経過した日、または研究結果の最終公表から3年を経過した日のいずれか遅い日までの期間が過ぎたのち、紙媒体はシュレッダー、その他の媒体に関しては適切な方法で廃棄する。

研究者等は、当該試料・情報の提供を行う者によって適切な手続がとられていること等を確認するとともに、当該試料・情報の提供に関する記録を作成する。研究責任者は、研究者等が作成した当該記録を当該研究の終了の報告から5年を経過した日、または研究結果の最終公表から3年を経過した日のいずれか遅い日までの期間保管する。研究責任者は、研究等の実施に係わる文書（申請書類の控え、病院長からの通知文書、各種申請書・報告書の控、研究対象者識別コードリスト、同意書、症例報告書等の控、その他データの信頼性を保証するのに必要な書類または記録など）を保存し、研究の終了の報告から5年を経過した日、または研究結果の最終公表から3年を経過した日のいずれか遅い日までの期間が過ぎたのち匿名化したまま廃棄する。

# 研究機関の長への報告内容及び方法

報告方法は各機関の規定により行う。

## 研究者等からの報告

研究者等は以下の場合、研究機関の長に報告をする。

- 研究に関連する情報の漏えい等、研究対象者等の人権を尊重する観点又は研究の実施上の観点から重大な懸念が生じた場合
- 研究の実施の適正性若しくは研究結果の信頼を損なう事実若しくは情報又は損なうおそれのある情報を得た場合

## 研究責任者からの報告

研究責任者は以下の場合、研究機関の長に報告をする。また、これに合わせて必要に応じて、研究の停止もしくは中止、および研究計画書の変更を検討する。

- 研究の倫理的妥当性若しくは科学的合理性を損なう事実若しくは情報又は損なうおそれのある情報であって研究の継続に影響を与えると考えられるものを得た場合
- 研究の実施の適正性若しくは研究結果の信頼を損なう事実若しくは情報又は損なうおそれのある情報を得た場合
- 研究の進捗状況
- 研究の実施に伴う有害事象の発生状況
- 研究を終了（中止の場合を含む。）した場合。結果概要を付けた研究終了報告書を作成し、報告する。

## 監査担当者からの報告

監査は行わないので、該当しない。

# 研究の資金源等研究に係る利益相反

利益相反に該当する企業はない。なお、日本薬剤師会 薬剤師職能振興研究助成事業に申請中である。

# 研究に関する情報公開の方法

本研究は研究開始前に、jRCT登録を行う。本研究の成果は、研究責任者に帰属するものとする。研究責任者、研究分担者および統計解析責任者が協議して著者を選出し、学会または論文にて報告する。研究対象者への結果の開示は行わない。

# 研究対象者等及びその関係者からの相談等への対応

研究対象者等及びその関係者からの相談等への対応窓口として、研究事務局が対応する。

# 代諾者等からインフォームド・コンセントを受ける場合の手続き

「人を対象とする生命科学・医学系研究に関する倫理指針」　ガイダンス　第4章　インフォームド・コンセント等　第9　代諾者等からインフォームド・コンセントを受ける手続等　２「代諾者等の選定方針」については、一般的には、次の①から③に掲げる者の中から、代諾者等を選定することを基本とする。

① （研究対象者が未成年者である場合）親権者又は未成年後見人

② 研究対象者の配偶者、父母、兄弟姉妹、子・孫、祖父母、同居の親族又はそれら近親者に準ずると考えられる者（未成年者を除く。）

③ 研究対象者の代理人（代理権を付与された任意後見人を含む。）

ただし、画一的に選定するのではなく、個々の研究対象者における状況、例えば、研究対象者とのパートナー関係や信頼関係等の精神的な共同関係のほか、場合によっては研究対象者に対する虐待の可能性等も考慮した上で、研究対象者の意思及び利益を代弁できると考えられる者が選定されることが望ましい。また、代諾者等からインフォームド・コンセントを受けたときは、当該代諾者と研究対象者との関係を示す記録を残すことも重要である。

# インフォームド・アセントを得る場合の手続き

本研究は18歳以上の成人が対象で患者本人の自由意思での参加を条件としているため、アセントは必要としない。

# 研究対象者等の経済的負担又は謝礼の旨及びその内容

本研究は、通常の診療範囲内で行われる。このため、研究対象者にかかる医療費は本人の健康保険を用いて行われる。本研究に参加する研究対象者に対し、交通費や謝金等の費用負担は行わない。

# 重篤な有害事象が発生した際の対応

プロトコール開始後84日以内に発症した有害事象については、その有害事象が改善するまで経過観察を行う。

緊急報告

1) 研究責任者／分担者は、重篤な有害事象が発生した場合、適切な処置を行う。研究分担者は、研究薬との因果関係を問わず、直ちに研究責任者に報告する。

2) 研究責任者は、当該重篤な有害事象について、直ちに医療機関の長に報告するとともに、研究薬提供者に通知する。また、必要に応じて効果安全性評価委員会に報告する。

3) 有害事象、研究薬との因果関係が否定できない有害事象に関する報告書式および手順は（各機関が定めた）「重篤な有害事象に関する手順書」に従う。

＜緊急時の連絡先＞

研究責任者：朝居　祐貴

三重中央医療センター　薬剤部

〒514-1101　三重県津市久居明神町2158-5

電話：059-259-1211 FAX ：059-259-0775 (薬剤部)

## 重篤な有害事象

有害事象のうち、次に掲げるいずれかに該当するものを「重篤な有害事象」とする。

1. 死に至るもの
2. 生命を脅かすもの
3. 治療のための入院又は入院期間の延長が必要となるもの
4. 永続的又は顕著な障害・機能不全に陥るもの
5. 子孫に先天異常を来すもの

「予測できない重篤な有害事象」とは、重篤な有害事象のうち、研究計画書、インフォームド・コンセントの説明文書等において記載されていないもの又は記載されていてもその性質若しくは重症度が記載内容と一致しないものをいう。

## 予測できる有害事象

カリウム制限に関する栄養指導により、低カリウム血症が生じる可能性が考えられる。

## 緊急報告

プロトコール開始後84日以内に発症した有害事象については、その有害事象が改善するまで経過観察を行う。

緊急報告

1) 研究責任者／分担者は、重篤な有害事象が発生した場合、適切な処置を行う。研究分担者は、研究薬との因果関係を問わず、直ちに研究責任者に報告する。

2) 研究責任者は、当該重篤な有害事象について、直ちに医療機関の長に報告するとともに、研究薬提供者に通知する。また、必要に応じて効果安全性評価委員会に報告する。

3) 有害事象、研究薬との因果関係が否定できない有害事象に関する報告書式および手順は（各機関が定めた）「重篤な有害事象に関する手順書」に従う。

＜緊急時の連絡先＞

研究責任者：朝居　祐貴

三重中央医療センター　薬剤部

〒514-1101　三重県津市久居明神町2158-5

電話：059-259-1211 FAX ：059-259-0775 (薬剤部)

# 研究によって生じた健康被害に対する補償の有無

研究対象者に本研究の参加に起因した健康被害が生じる可能性は無いと考えられるが、研究対象者に健康被害が生じた場合、研究責任者及び研究分担者は、適切な治療およびその他必要な措置を行う。この場合の治療等は保険診療として行い、自己負担分の医療費を研究対象者が支払う。

# 研究実施後における医療の提供に関する対応

本研究終了後の治療については規定しない。

# 研究対象者に係る研究結果の取扱い

研究対象者に対し、本研究の結果は非開示とする。本研究は探索的なものであり、結果の意義が現時点では確実性に欠けているため、研究対象者に知らせるには十分な意義がないためである。

# 研究に関する業務を委託する場合の内容及び委託先の監督方法

本研究では業務委託を行わないため、該当しない。

# 研究対象者から取得された試料・情報について研究対象者等から同意を受ける時点では特定されない将来の研究のために用いられる可能性又は他の研究機関に提供する可能性がある場合には、その旨と同意を受ける時点において想定される内容

データの正確性を見るために、論文の出版社からデータの提供を求められたり、全世界の研究者がデータを利用することへの提供（データシェアリング）を行ったりする可能性がある。この場合、データは、個人が特定できないよう加工したうえで提供し、対応表は提供しない。

# モニタリング及び監査を実施する場合には、その実施体制及び実施手順

## モニタリング

本研究は侵襲がないため、モニタリングを実施しない。

## 監査

本研究は侵襲がないため、監査を実施しない。

# その他

## 文献

1. 厚生労働省　腎疾患対策検討会報告書
2. Palmer BF, Carrero JJ, Clegg DJ, Colbert GB, Emmett M, Fishbane S, Hain DJ, Lerma E, Onuigbo M, Rastogi A, Roger SD, Spinowitz BS, Weir MR. Clinical Management of Hyperkalemia. Mayo Clin Proc. 2021 Mar;96(3):744-762.
3. Stevens PE, Levin A; Kidney Disease: Improving Global Outcomes Chronic Kidney Disease Guideline Development Work Group Members. Evaluation and management of chronic kidney disease: synopsis of the kidney disease: improving global outcomes 2012 clinical practice guideline. Ann Intern Med. 2013 Jun 4;158(11):825-830
4. Morris A, Krishnan N, Kimani PK, Lycett D. Effect of Dietary Potassium Restriction on Serum Potassium, Disease Progression, and Mortality in Chronic Kidney Disease: A Systematic Review and Meta-Analysis. J Ren Nutr. 2020 Jul;30(4):276-285.
5. Kurita N, Wakita T, Ishibashi Y, Fujimoto S, Yazawa M, Suzuki T, Koitabashi K, Yanagi M, Kawarazaki H, Green J, Fukuhara S, Shibagaki Y. Association between health-related hope and adherence to prescribed treatment in CKD patients: multicenter cross-sectional study. BMC Nephrol. 2020 Oct 31;21(1):453.
6. Ueda Y, Ookawara S, Ito K, Miyazawa H, Kaku Y, Hoshino T, Tabei K, Morishita Y. Changes in urinary potassium excretion in patients with chronic kidney disease. Kidney Res Clin Pract. 2016 Jun;35(2):78-83.
7. Saito Y, Yamamoto H, Nakajima H, Takahashi O, Komatsu Y. Incidence of and risk factors for newly diagnosed hyperkalemia after hospital discharge in non-dialysis-dependent CKD patients treated with RAS inhibitors. PLoS One. 2017 Sep 6;12(9):e0184402.
8. Okada H, Onda M, Shoji M, Sakane N, Nakagawa Y, Sozu T, Kitajima Y, Tsuyuki RT, Nakayama T. Effects of lifestyle advice provided by pharmacists on blood pressure: The COMmunity Pharmacists ASSist for Blood Pressure (COMPASS-BP) randomized trial. Biosci Trends. 2018 Jan 9;11(6):632-639.
9. Hiroshi Okada, Mitsuko Onda, Masaki Shoji, Kazuhiko Kotani, Takeo Nakayama, Yasushi Nakagawa, Naoki Sakane. Effects of Lifestyle Intervention Performed by Community Pharmacists on Glycemic Control in Patients with Type 2 Diabetes: The Community Pharmacists Assist (Compass) Project, a Pragmatic Cluster Randomized Trial. Pharmacology & Pharmacy. 2016 Mar,7(3):124-132.

## 別添

1．食事療法の指導：CKDに対する栄養指導に関する説明文書

2．カリウム含有食品の摂取についての意識調査のアンケート（介入前）（アンケート1）

3．カリウム含有食品の摂取についての意識調査のアンケート（介入後）（アンケート2）
