# Supplementary material for: A multicentral prospective cohort trial of a pharmacist-led nutritional intervention on serum potassium levels in outpatients with chronic kidney disease: The MieYaku-Chronic Kidney Disease project
Source: PLoS One. 2024 May 31;19(5):e0304479. doi: 10.1371/journal.pone.0304479 (PMC11142692; doi:10.1371/journal.pone.0304479)
Supplement: S1 Methods — (DOCX) [file pone.0304479.s005.docx]

**S1 Methods.**

**Questionnaire to Understand Attitude towards Consuming Potassium-Containing Foods**

**(Pre-intervention)**

This questionnaire has been approved by the Review Ethics Committee of Mie Chuo Medical Center (MCERB-202238)

Name:

*Please answer the questions as honestly as possible. The survey will be processed anonymously when it is tabulated.

**Question 1. Do you know that high level of serum potassium can be dangerous?**

- Very well
- Well
- Not that well
- Not at all

**Question 2. Do you know what foods have high potassium content?**

- Very well
- Well
- Not that well
- Not at all

**Question 3. Do you routinely regulate your consumption of foods with high potassium content?**

- Every time
- Sometimes
- Rare
- Not at all

**Question 4. Is it bothersome to be aware of your potassium intake?**

- Not at all
- Not much
- Slightly
- Extremely

**Questionnaire to Understand Attitude towards Consuming Potassium-Containing Foods**

**(Post-intervention)**

This questionnaire has been approved by the Review Ethics Committee of Mie Chuo Medical Center (MCERB-202238)

Name:

*Please answer as honestly as possible, as the survey will be processed anonymously when it is tabulated.

**Question 1. Do you know that high level of serum potassium can be dangerous?**

- Very well
- Well
- Not that well
- Not at all

**Question 2. Do you know what foods have high potassium content?**

- Very well
- Well
- Not that well
- Not at all

**Question 3. Do you routinely regulate your consumption of foods with high potassium content?**

- Every time
- Sometimes
- Rare
- Not at all

**Question 4. Is it bothersome to be aware of your potassium intake?**

- Not at all
- Not much
- Slightly
- Extremely

**Question 5. Has this guidance made you more careful about your intake of foods containing potassium?**

- Frequently
- Sometimes
- Not that much
- Not at all

**Question 6. Would you like to continue taking measures against consuming foods containing potassium in the future?**

□ Yes

□ Neutral

□ No

**Question 7. Please tell us if there is anything you noticed through this guidance. Please write your answer inside the box.**
