# Supplementary material for: A multicentral prospective cohort trial of a pharmacist-led nutritional intervention on serum potassium levels in outpatients with chronic kidney disease: The MieYaku-Chronic Kidney Disease project
Source: PLoS One. 2024 May 31;19(5):e0304479. doi: 10.1371/journal.pone.0304479 (PMC11142692; doi:10.1371/journal.pone.0304479)
Supplement: S1 Table — (DOCX) [file pone.0304479.s006.docx]

**S1 Table. Lecture contents in the MY-CKD project**

| Lecturer | Contents |
| --- | --- |
| Cardiologist  (20 min) | - - - Pathogenesis of hyperkalemia     - A physician’s perspective on hyperkalemia and dietary potassium restriction     - Current status of hyperkalemia treatment focused on outpatient care (treatment strategy, therapeutic agents) |
| Registered dietician  (20 min) | - - - Basic knowledge of dietary therapy for chronic kidney disease (CKD)   (Salt, protein, and potassium restriction and energy requirements)   - - - Dietary criteria by CKD Stage     - The current nutritional guidance document  1. Learning about foods with high potassium content. 2. Reducing potassium levels in foods by precooking. 3. Potassium restriction while maintaining dietary balance.    - - Attention to the potassium content of fruits. |
| Hospital pharmacist  (20 min) | - - - Drugs that induce hyperkalemia     - Importance of continuing renin-angiotensin system inhibitor and mineralocorticoid receptor antagonist after hyperkalemia in CKD     - A pharmacist’s perspective on hyperkalemia and dietary potassium restriction     - Details of the MY-CKD project |
