# Supplementary figures and images for: A multicentral prospective cohort trial of a pharmacist-led nutritional intervention on serum potassium levels in outpatients with chronic kidney disease: The MieYaku-Chronic Kidney Disease project
Source: PLoS One. 2024 May 31;19(5):e0304479. doi: 10.1371/journal.pone.0304479 (PMC11142692; doi:10.1371/journal.pone.0304479)

## Slide 1
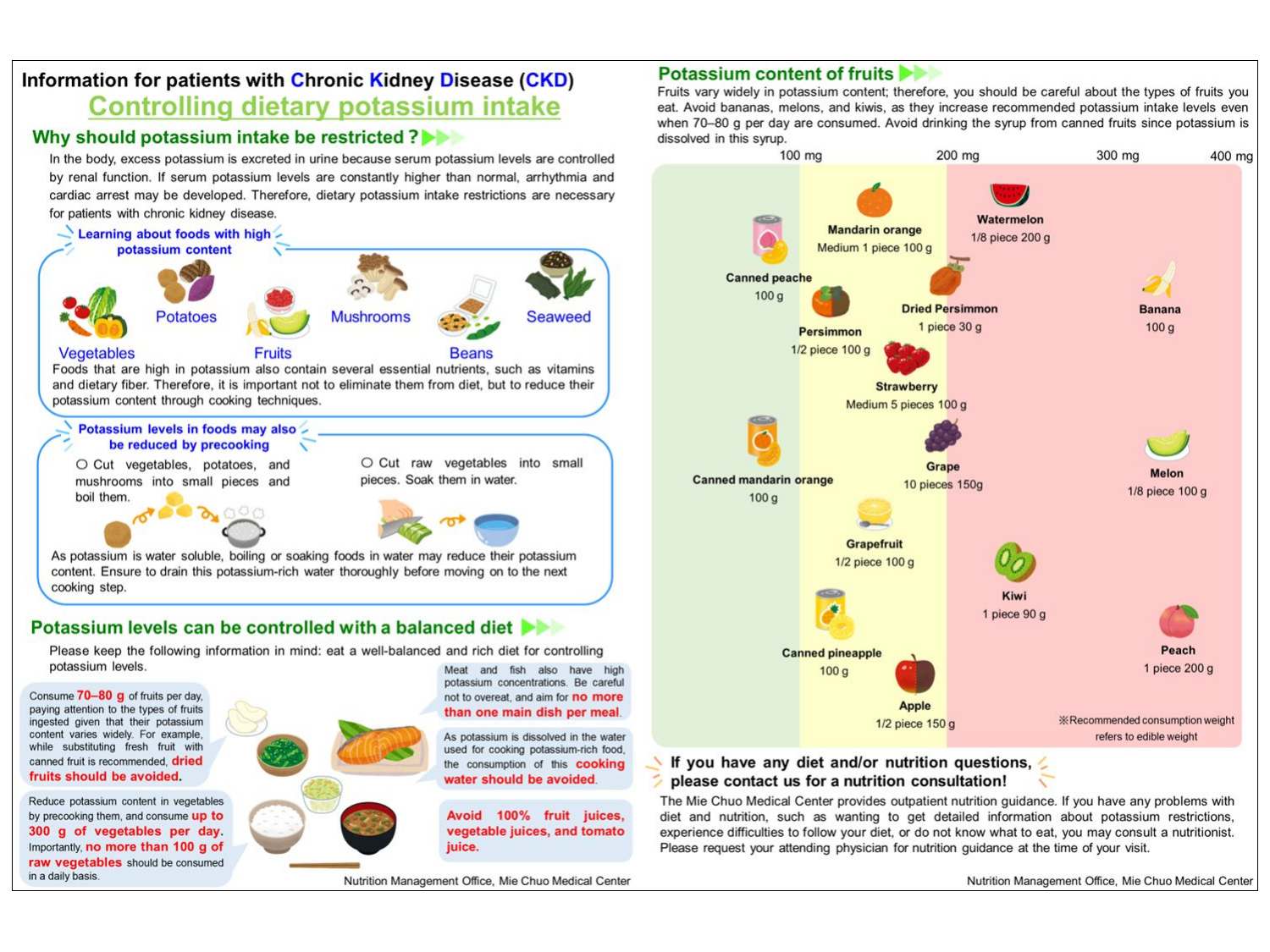

Supplement: S1 Fig — (PPTX) [file pone.0304479.s007.pptx]
