# Supplementary material for: A multicentral prospective cohort trial of a pharmacist-led nutritional intervention on serum potassium levels in outpatients with chronic kidney disease: The MieYaku-Chronic Kidney Disease project
Source: PLoS One. 2024 May 31;19(5):e0304479. doi: 10.1371/journal.pone.0304479 (PMC11142692; doi:10.1371/journal.pone.0304479)
Supplement: S2 Fig — Changes in (A) SBP and (B) DBP by dietary potassium restriction. The number of patients in pre-intervention and in post-intervention were 25 and 19, respectively. DBP, diastolic blood pressure. SBP, systolic blood pressure. CI, confidence interval. The differences in respective variables between pre- and post-intervention were compared using the Wilcoxon signed-rank test. (PPTX) [file pone.0304479.s008.pptx]

## Slide 1
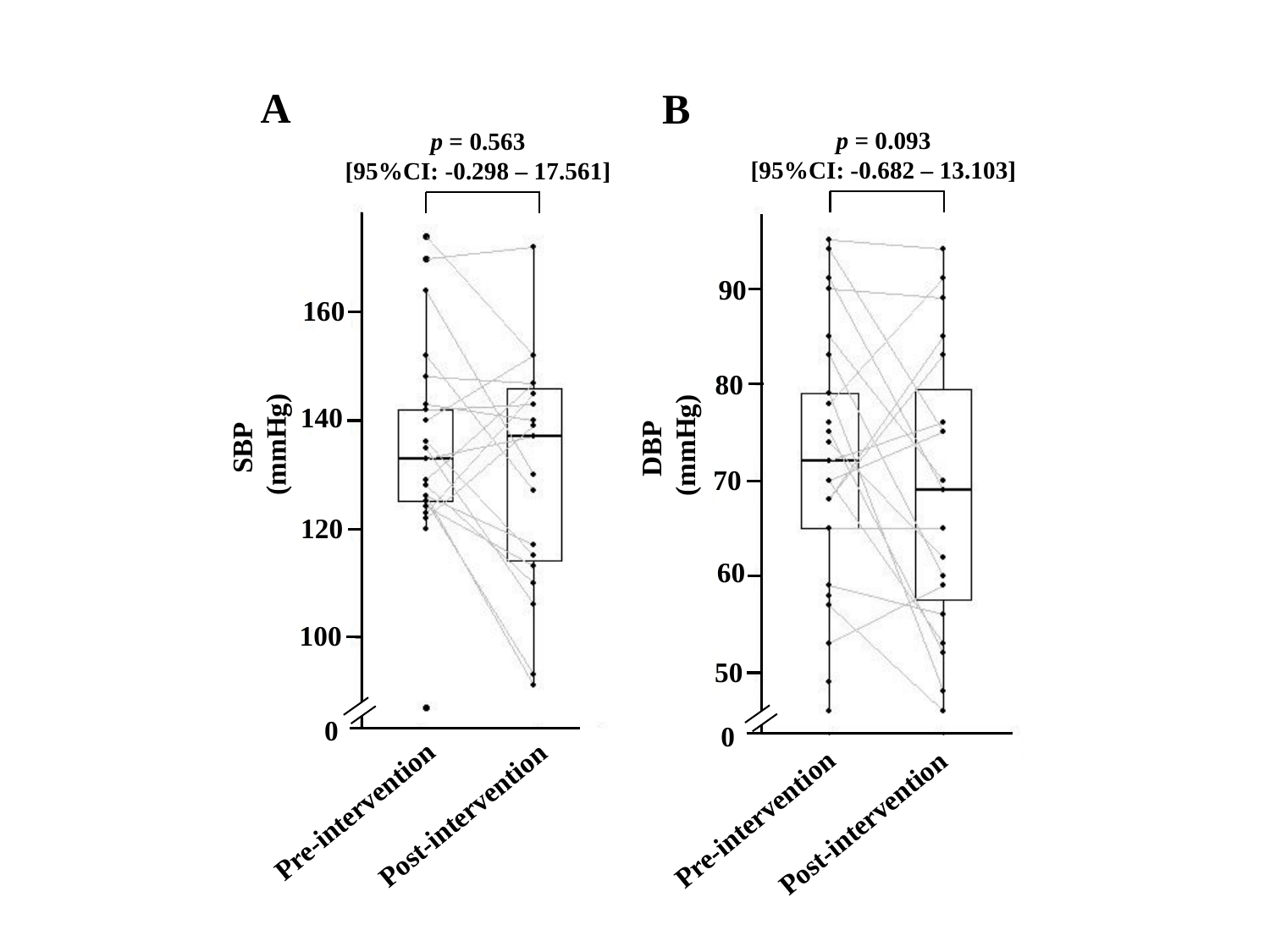

A
B
p = 0.093
[95%CI: -0.682 – 13.103]
p = 0.563
[95%CI: -0.298 – 17.561]
160
140
120
100
80
70
60
50
90
SBP
 (mmHg)
DBP
 (mmHg)
0
0
Pre-intervention
Post-intervention
Pre-intervention
Post-intervention
